# Genome Modeling System: A Knowledge Management Platform for Genomics

**DOI:** 10.1371/journal.pcbi.1004274

**Published:** 2015-07-09

**Authors:** Malachi Griffith, Obi L. Griffith, Scott M. Smith, Avinash Ramu, Matthew B. Callaway, Anthony M. Brummett, Michael J. Kiwala, Adam C. Coffman, Allison A. Regier, Ben J. Oberkfell, Gabriel E. Sanderson, Thomas P. Mooney, Nathaniel G. Nutter, Edward A. Belter, Feiyu Du, Robert L. Long, Travis E. Abbott, Ian T. Ferguson, David L. Morton, Mark M. Burnett, James V. Weible, Joshua B. Peck, Adam Dukes, Joshua F. McMichael, Justin T. Lolofie, Brian R. Derickson, Jasreet Hundal, Zachary L. Skidmore, Benjamin J. Ainscough, Nathan D. Dees, William S. Schierding, Cyriac Kandoth, Kyung H. Kim, Charles Lu, Christopher C. Harris, Nicole Maher, Christopher A. Maher, Vincent J. Magrini, Benjamin S. Abbott, Ken Chen, Eric Clark, Indraniel Das, Xian Fan, Amy E. Hawkins, Todd G. Hepler, Todd N. Wylie, Shawn M. Leonard, William E. Schroeder, Xiaoqi Shi, Lynn K. Carmichael, Matthew R. Weil, Richard W. Wohlstadter, Gary Stiehr, Michael D. McLellan, Craig S. Pohl, Christopher A. Miller, Daniel C. Koboldt, Jason R. Walker, James M. Eldred, David E. Larson, David J. Dooling, Li Ding, Elaine R. Mardis, Richard K. Wilson

**Affiliations:** 1 The Genome Institute, Washington University in St. Louis, St. Louis, Missouri, United States of America; 2 Department of Genetics, Washington University School of Medicine, St. Louis, Missouri, United States of America; 3 Department of Medicine, Washington University School of Medicine, St. Louis, Missouri, United States of America; 4 Siteman Cancer Center, Washington University School of Medicine, St. Louis, Missouri, United States of America; 5 Department of Molecular Microbiology, Washington University School of Medicine, St. Louis, Missouri, United States of America; University of Canterbury, NEW ZEALAND

## Abstract

In this work, we present the Genome Modeling System (GMS), an analysis information management system capable of executing automated genome analysis pipelines at a massive scale. The GMS framework provides detailed tracking of samples and data coupled with reliable and repeatable analysis pipelines. The GMS also serves as a platform for bioinformatics development, allowing a large team to collaborate on data analysis, or an individual researcher to leverage the work of others effectively within its data management system. Rather than separating ad-hoc analysis from rigorous, reproducible pipelines, the GMS promotes systematic integration between the two. As a demonstration of the GMS, we performed an integrated analysis of whole genome, exome and transcriptome sequencing data from a breast cancer cell line (HCC1395) and matched lymphoblastoid line (HCC1395BL). These data are available for users to test the software, complete tutorials and develop novel GMS pipeline configurations. The GMS is available at https://github.com/genome/gms.

*This is a PLOS Computational Biology Software Article.*

## Introduction

The increasing sequence data output of massively parallel sequencing platforms [1] has allowed the application of sequencing to an incredible diversity of research projects in the biological, genomic, and medical fields [2–6]. These technologies have inundated their adopters with petabytes of data, outpacing their ability to effectively manage and analyze the data. A rapid proliferation of tools and resources to analyze these data [7–10] complicates the creation and maintenance of analysis pipelines.

**Fig 1:**
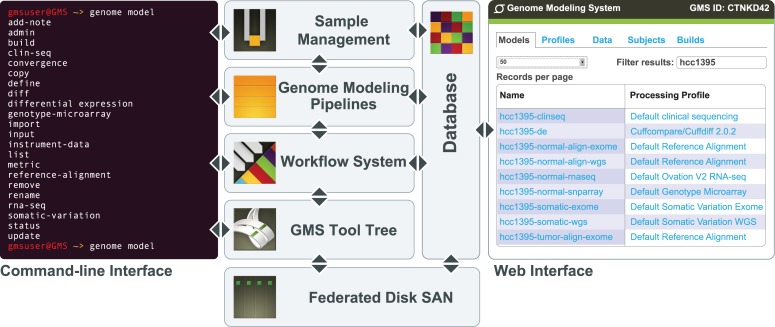
Overview of the GMS. The genome modeling system (GMS) is implemented to use a federated disk SAN, with meta-data stored in a PostgreSQL relational database. Sample management tools allow the import of new samples and instrument data. Data are then processed through various analysis pipelines (e.g., reference alignment, somatic variation detection, etc.) that in turn are managed and monitored by a workflow system (Box 1). Stand-alone GMS tools, not part of automated pipelines, are available through a common tool tree. Most components of the system can be accessed through an Ubuntu Linux command-line interface or Ruby-on-Rails web interface.

The GMS is the core analysis system at The Genome Institute (TGI) of Washington University, processing terabases of genomic data and proving integral to a wide variety of large- and small-scale sequencing projects (Fig 1). Pipelines implemented within the GMS include reference sequence alignment, germline variant detection, somatic variant detection, RNA-seq (expression, novel transcript detection, and fusion detection), differential expression, and others (Table 1). The GMS also includes an integration, annotation, and interpretation pipeline, ‘MedSeq’, which attempts to converge all single-subject data into a form suitable for identification of clinically actionable events [11]. A typical genome analysis using the GMS might start from any combination of whole-genome, exome or RNA-seq data and produce alignments against a reference genome, somatic variant calls including single nucleotide variants (SNVs), structural variants (SVs), copy-number variants (CNVs), transcript expression levels, RNA fusion predictions, and more. To date, the GMS has been used to process >4,800 human whole genome samples, >40,000 exomes and >1,400 transcriptomes for a total of >700 terabases of sequence data (Table 2).

**Table 1:** Major GMS pipelines. A brief description of each analysis pipeline tested for initial release of the GMS.

Pipeline	Description	Products
Genotype Microarray	Performs genotype calling on SNP array data against a reference sequence.	SNVs BED file.
Reference Alignment	Performs alignment and variant detection for reads from a single sample. Works with WGS data and capture data.	BAM file of aligned reads, VCF files and BED files for germline SNVs, Indels, SVs, and CNVs. Reports on coverage.
Somatic Variation	Performs tumor/normal variant detection. Extends reference alignment with somatic evaluation, LOH analysis, annotation and prioritization. Works with WGS data and capture data.	VCF files and BED files for somatic SNVs, Indels, SVs, and CNVs.
RNA-seq	Uses Bowtie/TopHat/Cufflinks to assemble transcripts and estimate abundance, alternative splicing, alternative promoter usage, etc. Also uses various tools to perform comprehensive quality and coverage analysis of RNA-seq libraries	Spliced alignment BAM, FPKM expression, digital expression, fusion detection, etc.
Differential Expression	Combines results from a pair of RNA-seq builds and performs differential expression analysis.	CuffDiff and CummeRbund output.
Med Seq (aka Clin Seq)	Integrates data from WGS, exome and transcriptome sequencing of a single patient’s tumor. Visualization and annotation of somatic events. Prioritization of somatic events by relevance to cancer biology and therapeutic decision making.	Approximately 2,000 files, including: spreadsheets of ranked and annotated variants, drug-gene interactions, Circos plots, copy number images, mutation diagrams, etc.

**Table 2:** Data processed by the GMS. A brief summary of data processed by use of the GMS at The Genome Institute of Washington University School of Medicine in St. Louis (as of October 2014).

Metric	Human	Non-human	Total
WGS cases (samples)	2,517 (4,349)	355 (534)	2,872 (4,883)
Exome/targeted cases (samples)	30,343 (35,366)	6,027 (8,270)	36,370 (43,636)
RNA/cDNA cases (samples)	375 (555 samples)	711 (855 samples)	1,086 (1,410)
Bp of Illumina NGS reads	622 terabases	82 terabases	704 terabases

**Box 1:** Terminology for the Genome Modeling System. Brief descriptions of critical objects in the Genome Modeling System.

Term	Definition
Subject	The entities around which analysis occurs. Exist at multiple levels of granularity. For example, an individual, a cohort, a sample from an individual, or even a species. Anything that can be described abstractly as “having a genome”. When the subject is a human patient, use of the GMS will normally require appropriate ethics review and informed consent of the patient. Related documentation will be linked to analyses via an anonymized unique patient number (UPN) stored in the GMS subject database table along with additional metadata.
Model	The basic unit of analysis. Each model represents one state of belief about the sequence and features of a given subject. Multiple models can be made of the same subject, with different processing profiles, and/or different input data used as evidence.
Pipeline	Each type of model defines a distinct analysis pipeline. The definition includes a specification for inputs and parameters to each model, as well as logic to construct a workflow to build results given specific values for those inputs and parameters.
Processing Profile	A reusable collection of parameters describing how to build a model of a particular type/pipeline. Each is a complete computational method specification, including exact tool names and versions, as well as sufficient logic to determine the precise workflow. All models with the same processing profile have been processed the same way, though input data may vary.
Build	One attempt to execute the required workflow for a model, given its inputs. The last complete build for a model represents the current “state” of the model. While models can be updated, the information content in each build is a static snapshot of results.
Instrument Data	A unit of data from a sequencer, microarray instrument, or other device, used as primary input to the GMS. Illumina data, for instance, produces one unit of instrument data per flow cell, lane, and index sequence. It is typically associated with a file of reads, and a collection of metrics.
Software Result	A reusable intermediate result made by the build process. When the exact same process is to occur a second time on the same inputs with the same parameters, the software result produced the first time is detected. The GMS uses these to prevent redundant work, and expedite processing after minor analysis protocol changes.
Disk Allocation	A record of a slice of disk being allocated to a given owner. Builds, software results, and instrument data are owners of disk allocations.
Workflow	A graph of steps, and the data flow between those steps. A workflow is generated for each attempt to build a model. Individual steps may also define subordinate workflows, leading to a nested graph of tasks to accomplish the analysis goal.

As a demonstration of the GMS, we describe a complete integrated analysis of whole genome, exome and transcriptome sequencing of a breast cancer cell line (HCC1395) and a matched lymphoblastoid cell line (HCC1395 BL). The complete dataset is publicly available (https://xfer.genome.wustl.edu/gxfer1/project/gms/). The GMS is available as open-source software with installation instructions at http://github.com/genome/gms. Once installed, users can run tutorials, reproduce the results from this publication, and test novel GMS pipeline configurations. This ability to replicate and iteratively improve upon large and complex genome analyses will allow researchers to more easily manage the immense challenges of modern large-scale sequence analysis.

## Design and Implementation

To address challenges of scale, tracking, optimization, and reproducibility, we have developed an analysis information management system called the Genome Modeling System (GMS). The GMS tracks analysis processing steps while also managing project and sample information (Fig 1). It records sufficient detail in a relational database about each computational experiment to reproduce it entirely from metadata. The information is stored and indexed to enable free-form search. Results are also stored in standard formats (e.g. BAM [12], Variant Call Format (VCF) [13], Tabix [14]) with a record of the methods and inputs that produced them. The system can automatically bypass regeneration of intermediate results when those results have already been created as part of another process, hence saving immense amounts of disk and compute resources. It can also automatically aggregate data across samples within a project to provide high-level overviews of analysis status and results. Finally, the GMS facilitates the comparison of analysis results. A user can compare the output of several analysis pipelines utilizing different alignment parameters, variant callers, filters, and many more variables. The ultimate goal of the GMS is to make data management, analysis, and integration more accessible at scale.

The GMS is driven by a flexible command-line interface and a web interface for monitoring. The web interface includes search capability for all of the major entities stored in the system, with free-form search based on full-text matching. The command-line interface is built around a single command, "genome", which offers a multi-level tree of sub-commands. These commands give access to all of the tools and data in the system (S1 Fig). The top level of the command tree allows interaction with instrument data, samples, and analysis results that are stored in the database (Box 1), including the ability to create, list, update and delete them (Box 2).

**Box 2. Example Usage**

Simplified examples of command-line usage are provided for illustrative purposes (see the tutorials at http://github.com/genome/gms/wiki for fully functional examples.) First, samples are listed for a given patient/subject by anonymized identifier (patient1). All commands that work with database entities support an expression syntax that allows items to be selected from the database by ID, or by other characteristics. Next, specific units of instrument data are examined for the first sample (S1). Processing profiles are listed for the reference alignment pipeline. A model is then defined for the first sample (S1) using the second processing profile (P2). Instrument data (I1, I2, and I3) are assigned as an input. The build process is then initiated, recording the new build (B1) uniquely in the database, and starting jobs on the compute cluster. A *build view* command is then used to monitor the steps involved in the build workflow, examine logs and check run times. The results are accessible as files, for downstream analysis with additional metrics also in the database.

> **genome sample list “individual.common_name = patient1”**

 id common_name individual.common_name

 S1 tumor patient1

 S2 normal patient1

 S3 relapse patient1

 > **genome instrument-data list sample.id = S1**

 id flow_cell_id lane index_sequence sample.id

 I1 ABC123 1 <NULL> S1

 I2 ABC123 2 AGCT S1

 I3 ABC123 2 TCAG S1

 > **genome processing-profile list reference-alignment**

 id type_name name

 P1 reference alignment BWA 0.5.9 and samtools

 P2 reference alignment BWA-MEM 0.7.2a and Gatk

 > **genome model define reference-alignment--subject id=S1--processing-profile id=P2--name=“TST1 tumor”**

 defined genome model M1

 > **genome model input add instrument_data id=M1 "flow_cell_id='ABC123' and lane in [1,2]"**

 assigned instrument data I1, I2 and I3 to model M1

 > **genome model build start id=M1**

 new build B1 started for model M1 with data directory at /opt/gms/MYSYS1/fs/model_data/M1/buildB1/

 > **genome model build view id=B1**

 
**> cd /opt/gms/XYZ123/fs/model_data/M1/buildB1**

 
**> samtools view alignment_results/12345.bam**

### Tool Tree and Application Programming Interface (API)

At the core of the GMS is a "tool tree", into which bioinformaticians collaboratively add components to build up a software library of computational tools and methods for their organization. Tools are accessible through the “genome tools” command, aliased by “gmt”. Adding a component to the tool tree requires writing a command class by following detailed documentation aimed at prospective developers with basic programming skills. Tools work directly on simple files, and provide fast access to the small scripts an analyst typically creates during their daily bioinformatics work. These components can evolve into complex systems, gradually, and only as needed. Additional features such as tests, documentation, and compositional pieces can be added incrementally. A low barrier to initial entry is essential to keeping the tool tree at the center of method development. S2 Fig shows an example tool, its position in the tree, its source code, and the help text generated from metadata in the software module.

Any analyst using the system automatically works in their own software ‘sandbox’, allowing private changes to any part of the system. Tools and pipelines can be added without outside registration and function for that user as though the user had deployed the tool at large in the GMS. The analyst can then push their changes to be used more broadly in the organization, or share them with the community at large. The tool tree packaged with the GMS contains over 1,500 bioinformatics components organized into 150 categories. These include tools to work with established bioinformatics software such as BWA [15], TopHat [16], Blat [17], HTSeq [18], and liftOver [19], as well as in-house tools such as DGIdb [11].

### Models

**Fig 2:**
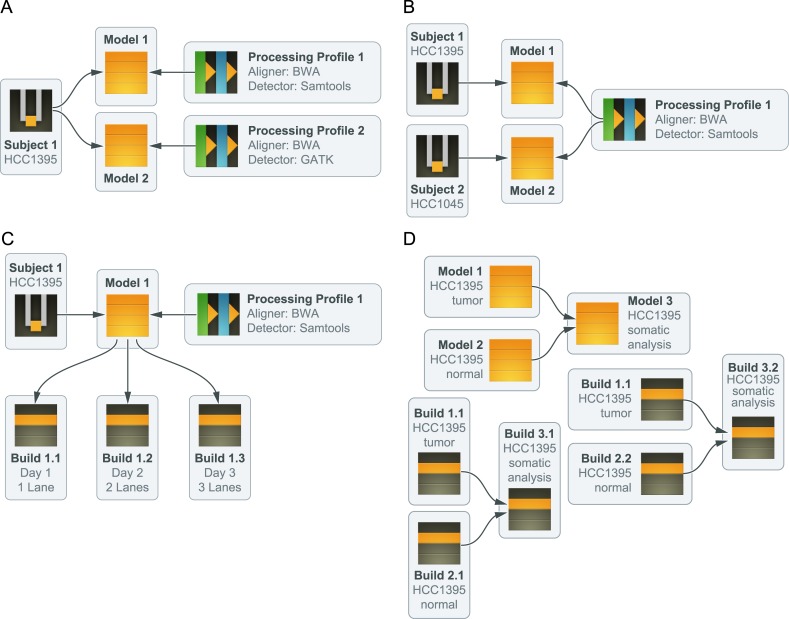
Key concepts of the GMS. The genome modeling system is architected around the idea of a ‘genome model’. The following vignettes illustrate key concepts integral to these models: (**A**) A subject can be modeled multiple times, possibly each with distinct ‘processing profiles’. For example, two different models can be defined for the HCC1395 genome using the ‘reference alignment’ pipeline. In Model 1, the processing profile specifies the use of BWA for alignment and Samtools for variant detection. In Model 2, Bowtie2 and GATK are used for these steps instead. (**B**) A given processing profile can be used across a group of models, ensuring, for instance, that all subjects in a cohort are processed in similar ways. In this example, two different cell line genomes (HCC1395 and XY2123) have models defined of the exact same type, using the processing profile with BWA/Samtools specified. (**C**) A model has no results until a build is generated. If the model is updated to have new inputs, a new build is required. Builds are immutable snapshots of modeling pipeline results. In this example, the HCC1395 genome has a reference alignment model again making use of the BWA/Samtools profile. However, as new instrument data becomes available, new builds are constructed to reflect the most complete data. (**D**) When models are used as inputs for other models, the last complete build for the input model is used as an input for the downstream build. In this example, both tumor and normal genomes are available for an individual (in this case HCC1395). Reference alignment models are built for each sample and then both are used as inputs for a third ‘somatic variation’ model. In reality, it is the underlying data in the reference alignment builds that are used to create a somatic variation build, identifying all variants that are thought to be tumor specific.

The central metaphor for analysis products in the GMS is the ‘genome model’ (Fig 2A). Each model represents one state of belief about the sequence data and features of a given subject. Multiple approaches to arrive at a conclusion for the same subject will be represented as multiple models in the system, each with a different ‘processing profile’ to describe the methods in precise computational terms (Box 1).

#### Processing Profiles

Each *processing profile* describes in detail how an analysis should occur. It does so in a declarative fashion. A processing profile embeds exact tool versions and parameters, such that two models built with the same processing profile, inputs, and GMS software version will have identical results (Box 1). This also allows all subjects in a given cohort to be processed in the same way if consistency is desired (Fig 2B). Each pipeline in the GMS has a collection of processing profiles that describe each of the ways the pipeline can be run. Each profile is given an identifier in the database, and new processing profiles can be created to apply different computational approaches, either by constructing a new processing profile from scratch, or by copying an existing one, and adjusting the parameters. For example, a user might decide to detect variants with a different tool, or to apply read trimming before alignment. This system allows an analyst to experiment with different methods almost as easily as describing those methods in a conversation. Hence, complex workflows do not require manual construction and can be computationally derived from a declarative specification (Box 1).

### Subjects

The subject of a model determines which genome it intends to examine, much as the processing profile determines how it will be examined (Box 1). The subject of a model is sometimes a particular individual, but is more often a specific sample from some individual. In germline analysis of human disease, one model will be created for each individual, and a model group or population model used to summarize across a cohort. In cancer analysis, one model will be made for the genome of the tumor, and another for the genome of a matched normal, with a third performing the comparison between the two. The MedSeq (aka ClinSeq) models target the individual in general, taking other models as inputs, each with more specific subjects relating to tumor or normal DNA or RNA. It should be noted that while this work primarily describes a computational/analysis platform, when the GMS is applied to real patients its use will normally require appropriate IRB review and informed consent as per the requirements of the user’s jurisdiction and institutional policies.

### Inputs

In the most basic case, a model’s inputs will include instrument data. The system can handle sequence data generated by sequencing instruments from Illumina (GAII, HiSeq 2000, HiSeq 2500, and MiSeq), Pacific Biosciences and Ion Torrent. In addition to sequence data, microarray data can also be supplied as input. Models often require reference sequences, annotation, or lists of regions of interest, depending on the model type. The subject of a model may limit what inputs can be assigned, ensuring that assigned reads are actually from the subject in question, and that an input reference sequence applies to the species of the subject.

### Builds (Performing Analysis)

Once a model is defined, it is ‘built’. Each attempt to build a model launches a ‘workflow’ on the compute cluster, and adds a record of that build to the database for the model in question to track processing. The workflow management process is described below.

A model may be built multiple times. This occurs typically when new instrument data are assigned (Fig 2C), a new reference sequence becomes available, or new gene annotations are published and imported into the GMS. It also occurs when processing errors cause a build to fail. A complete build of a model represents a collection of results of the processing specified by the model (e.g. germline variants discovered in blood, somatic variants discovered in a tumor, novel transcripts expressed in a tissue, genes differentially expressed between conditions, etc.). The disk space allocated for the build contains VCF files for variants, BAM files for alignments, and a variety of other reports and images. At a logical level, the bundle of data produced during the build process can be interrogated by build ID to query the state of the genome in question. The resulting model can subsequently be used as an input to other models. In this case, each build of the downstream model records the current build of the upstream model as an input (Fig 2D). Because builds are conceptually immutable, every data product in the GMS can be traced back to original sequencing instrument data, and can be reproduced reliably.

### Pipelines

**Fig 3:**
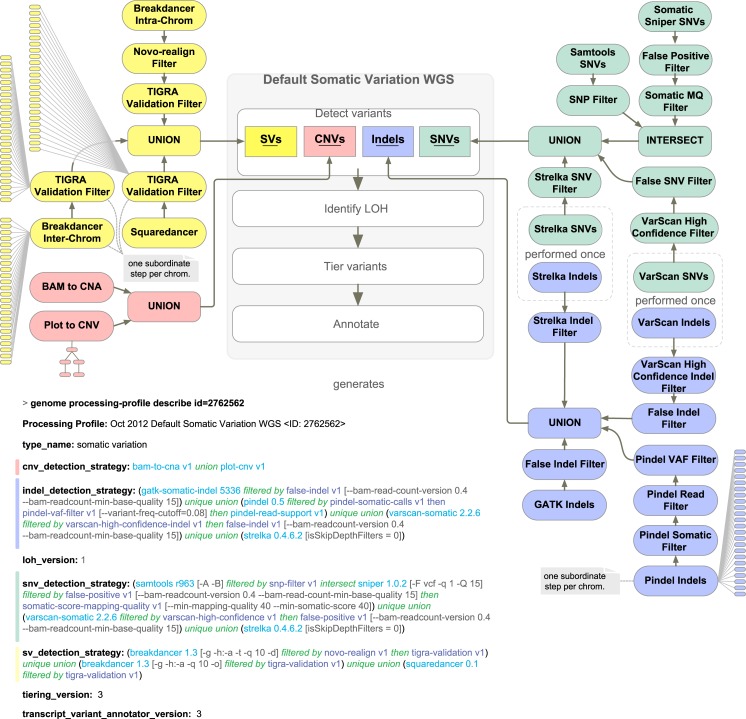
Somatic variation processing profile and workflow. To illustrate key GMS concepts, the processing profiles and workflow for the somatic variation pipeline are shown. Abbreviations: copy number variant (CNV), copy number amplification (CNA), genome analysis tool kit (GATK), insertion/deletion (Indel), loss of heterozygosity (LOH), mapping quality (MQ), single nucleotide variant (SNV), structural variant (SV), variant allele frequency (VAF).

Each type of model defines a distinct analysis pipeline, including a specification for inputs and parameters to be supplied when models are created as well as logic to construct the workflow and to parse build results. Adding new pipelines requires writing a software module to describe the new sub-type of model. The simplest pipelines are no more complicated than a small script, and the most complicated have an elaborate graph of steps, each with distinct processing requirements. As an example of the latter, Fig 3 details the workflow of the Somatic Variation pipeline. In most cases, the exact tools and versions to use for any given stage in a pipeline are configurable in the processing profile. Some fields are specific thresholds or other simple parameters. In many cases, however, the processing profile fields contain expressions that can be expanded into a sub-workflow. For example, variant detection is specified with four fields. The ‘sv_detection_strategy’ shown in Fig 3 involves a pair of variant detectors, one of which is run twice in different modes, and a series of different filters and intersection logic for the results. The entire process will create a sub-workflow based on the specification shown. One of the detectors defines another sub-workflow to process data by chromosome, and another to look for inter-chromosomal translocations. Some of the filters simply examine metrics, while others perform realignment. Other filters perform small *de novo* assemblies to validate structural variant predictions *in silico*. This example illustrates how arbitrarily complex workflows can be specified by creation of custom processing profiles.

For additional details on design and implementation, refer to the Supplementary Methods (S1 Text).

## Results

The GMS has been used at The Genome Institute to analyze a large number of genomes in both clinical and discovery contexts (Table 2). For example, the GMS has been instrumental for the analysis of nearly all The Cancer Genome Atlas (TCGA), Pediatric Cancer Genome Project (PCGP) [20], and other large-scale cancer genomics efforts at the Genome Institute, helping to map the landscapes of endometrial carcinomas [21], acute myeloid leukemias [22], pediatric low-grade gliomas [23], breast cancers [24], non-small-cell lung cancers [25], colon and rectal cancers [26], and ovarian cancers [27], among others. The GMS has also been used to assemble new genomes [28, 29], conduct studies of common [30] and rare disease [31, 32], track the evolution of viruses [33], and characterize the human microbiome [34, 35].

**Fig 4:**
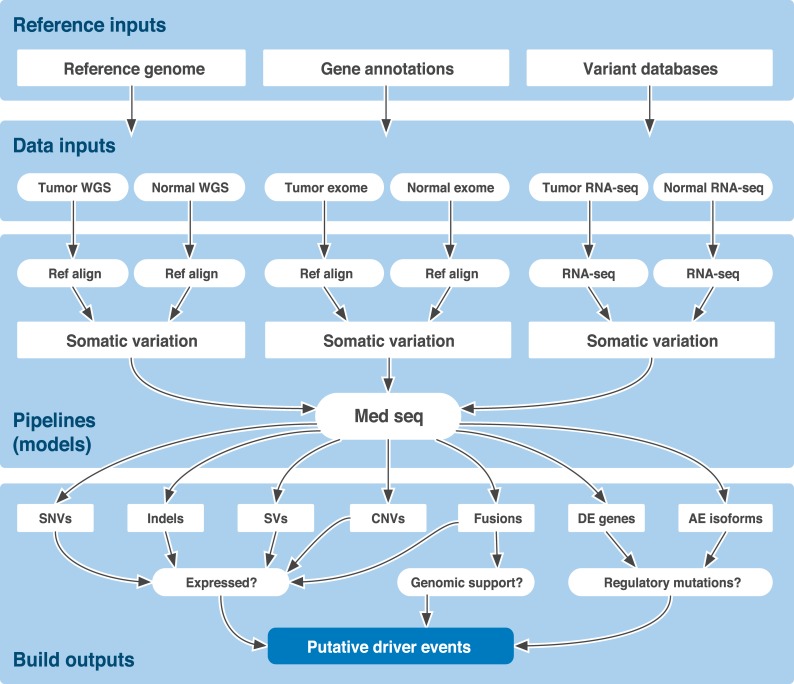
HCC1395 (“TST1”) example input, models, and outputs. A test dataset for the HCC1395 cell line is provided with the GMS software to allow testing of software installation, and facilitate further development. It is also used to illustrate much of the current functionality of the GMS. HCC1395 tumor and the corresponding HCC1395BL ‘normal’ cell line DNA and RNA samples were sequenced by whole genome, exome, and RNA-seq methods producing six sets of instrument data for input to various GMS pipelines. Additional required inputs for the pipelines include a reference genome (e.g., GRCh37), gene annotations (e.g., Ensembl 67_37l), and variant databases (e.g., dbSNP37). Different versions (processing profiles) of the reference alignment were used to align WGS and exome DNA reads to the reference genome. A separate RNA-seq pipeline similarly aligns RNA reads. Alternate versions of the somatic variation pipeline are used to call various types of variants from exome and WGS data by comparing tumor and normal reference alignments. A differential expression pipeline identifies significantly altered transcript expression levels by comparing the tumor and normal RNA-seq alignments. Finally, the MedSeq pipeline summarizes all upstream pipelines into a single convenient result set. This includes a multitude of reports and visualizations for single nucleotide variants (SNVs), Indels (insertions and deletions), SVs (structural variants), CNVs (copy number variations), transcript fusions, differentially expressed genes, alternatively expressed isoforms, and much more. Data types are further integrated to, for example, identify which variants at the DNA level are expressed at the RNA level and which events affect known cancer driver genes or druggable targets.

As a demonstration we applied the GMS to an integrated analysis of whole genome (WGS), exome, and transcriptome sequencing of a breast cancer cell line (HCC1395) and matched ‘normal’ lymphoblastoid cell line (HCC1395/BL [36]). The latter cell line is matched to the same individual (also referred to as ‘TST1’ below). A total of 10 lanes of HiSeq 2000 (v3 chemistry) sequence data consisting of ~1.8 billion 2x100bp reads were produced for HCC1395 and HCC1395/BL. Whole genome sequencing, exome sequencing and RNA-seq were performed as described previously ([25, 37] and S1 Text). HCC1395 and HCC1395/BL were sequenced to average coverage levels of 56x (WGS)/155x (exome) and 31X (WGS)/124x (exome), respectively. RNA sequencing achieved 20x coverage of >50% of known junctions for 8,640 genes for HCC1395 and 9,437 genes for HCC1395/BL respectively. Complete quality and coverage statistics from automatically generated GMS reports were summarized for WGS (S1 Table), exome (S2 Table) and RNA-seq data (S3 Table). Genotypes determined from whole genome NGS data were compared to those determined by Illumina Infinium microarrays and an overall concordance of 98.7% and 99.6% was observed for the tumor and normal calls respectively. Fig 4 shows the collection of models and their forward progression through the HCC1395 analysis. All of the following statistics and figures were drawn directly from automated output of the following GMS pipelines: ‘genotype microarray’, ‘reference alignment’, ‘somatic variation’, ‘rna seq’, ‘differential expression’ and ‘med seq’ (aka ‘clin seq’). Distinct somatic-variation processing profiles were used for the whole genome and exome data sets. The HCC1395 data is made publicly available (https://xfer.genome.wustl.edu/gxfer1/project/gms/) to allow GMS end users to reproduce this analysis. All tutorials and examples in the online documentation are based on these data. For complete details on how these data were generated, refer to the Supplementary Methods (S1 Text).

**Fig 5:**
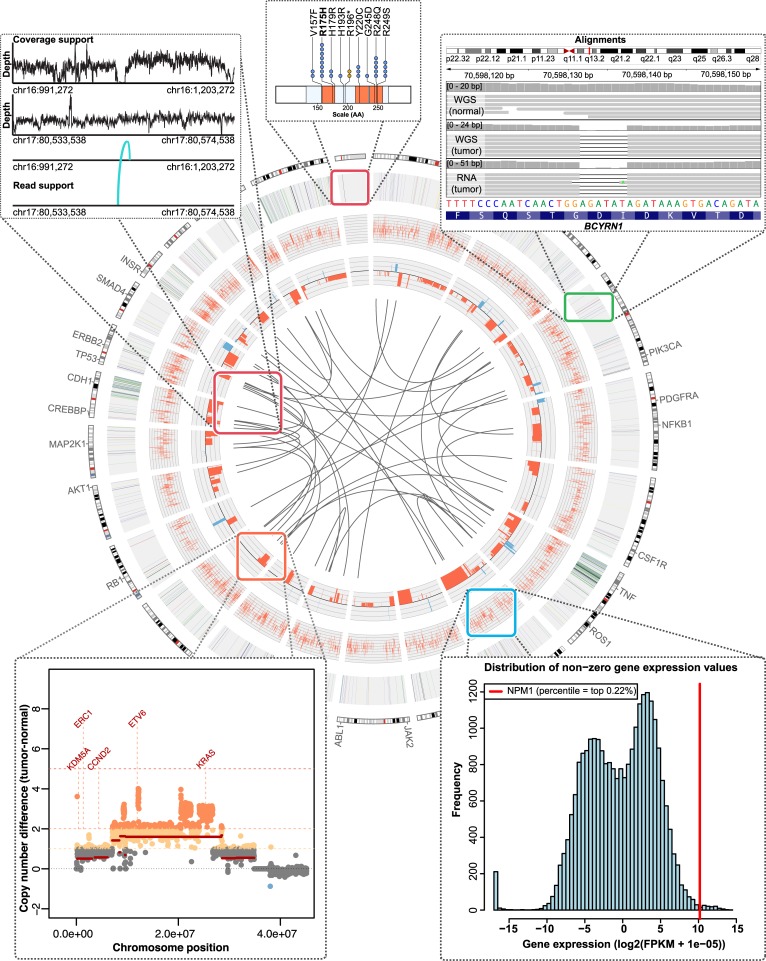
Circos plot of HCC1395 tumor/normal comparison. Circos is a popular tool for summarizing genomic events in a tumor genome. This is just one of many automatically generated visualizations made possible by the GMS. In this example, the WGS, exome and RNA-seq data for HCC1395 are displayed in several tracks along with additional visualizations illustrating individual events. Moving inwards, SNVs and Indels are plotted on the outermost track, then highly expressed genes, CNVs, and finally chromosomal translocations at the center. For events predicted to affect protein coding genes, additional plots are auto-generated to display the mutation position relative to protein domains and previously reported mutations from the Cosmic database, as illustrated in the topmost plot. Moving clockwise, a screenshot of IGV demonstrates one of the somatic deletions identified. IGV XML sessions are automatically generated to allow rapid manual review of all predicted events. Next, a histogram illustrates the expression of a single highly expressed gene relative to the distribution of expression for all genes. Then, a CNV plot is shown for an amplified portion of one chromosome. Finally, the coverage and supporting reads for a chromosomal translocation are depicted.

Examples of key data produced by GMS analysis pipelines are summarized in Fig 5 and provided in the supplementary materials (S3–S11 Figs and S1–S7 Data). S3 Fig shows the copy-number analysis for WGS data of tumor and normal, and one example of a selected CNV amplification on chromosome 12. Amplifications of known cancer-related genes such as *KRAS* and *ETV6* are automatically labeled. Unsurprising for a cell line, the ploidy of HCC1395 is highly aberrant with large-scale amplifications and deletions evident on all chromosomes. The highly copy number altered genome of HCC1395 complicates accurate somatic event detection. The GMS facilitates integrated use of multiple variant detectors to take advantage of the varying strengths of each. A breakdown of somatic SNV calls by algorithm, and the results from manual review by the Integrative Genomics Viewer [38] (IGV) of those variants are provided in S4 Fig. A high mutation rate was observed in HCC1395 (47 mutations/Mbp), likely due to the large number of cell divisions in multiple cell line passages and to the mutations we detected in DNA damage surveillance/DNA repair genes, including: *MSH6*, *TP53*, *ATRX*, *BRCA2*, *MSH5*, and *POLH*. Selected lists of cancer genes, curated by the Genome Institute from a variety of sources and released with this system, are intersected with high-confidence variant calls (S5 Fig). This allows rapid sorting of mutated gene lists according to those identified as previously mutated in Cosmic [39] or belonging to cancer-relevant gene categories according to GO [40], the cancer gene census [41], Entrez [42], and other sources. A selection of these mutations and associated annotations are provided in S4 Table. When variants affect protein coding genes, ‘lolliplot’ mutation diagrams of the predicted amino acid effect are automatically generated, showing the location of the mutation(s) relative to known domains and to the known mutational landscape according to Cosmic (S6 Fig). For example, in HCC1395 we observed a potentially novel mutation in *BRCA2* as well as mutations in *NCOR2* and *TP53* that occur at previously observed hotspots. A complete list of all somatic SNVs detected in HCC1395 is provided in S1 Data. S7 Fig shows a *TAF1* deletion, with an image of the reads in all five of the samples, and a clear visualization of the variant in the tumor DNA, WGS and exome, as well as tumor RNA and a compelling absence of such variation in any of the normal samples. The MedSeq pipeline automatically creates XML session files to allow rapid loading of all necessary BAM alignment files, BED files of variant calls and the appropriate reference genome in the IGV browser from which this screenshot was produced. We find this particularly useful for putative Indels where a high false positive rate is common. A companion ‘lolliplot’ shows that this is an in-frame deletion of *TAF1*. The complete list of predicted Indels in HCC1395 is provided in S2 Data. S8 Fig shows coverage and variant allele frequency (VAF) data for tumor and normal samples and contrasts the values derived from the WGS, exome and RNA-seq data. The complete list of predicted CNV events is provided as S3 Data. S9 Fig shows a list of putative ORF-maintaining gene fusions detected with the SV pipeline using BreakDancer [43] and CREST [44] (aka ‘SquareDancer’). A ‘pairoscope’ plot illustrates the supporting reads for one of these potential fusions between *PRTG* and *MALT1* on chromosomes 15 and 18 (S9 Fig). The complete list of predicted SVs from BreakDancer is provided as S4 Data. A complete set of gene expression and exon splicing results are provided as S5 Data and S6 Data. The complete list of RNA gene fusion predictions from ChimeraScan [45] is provided as S7 Data. S10 Fig shows a clonality plot, demonstrating a very pure and homogenous sample as evidenced by a single clear distribution of variant allele frequencies (VAF) centered almost exactly at 50% VAF, as expected for heterozygous variants. S11 Fig illustrates a small sample of the many graphs automatically generated to interpret RNA-seq results. Library quality can be assessed by observed insert size distribution (S11A Fig) and end bias (S11B Fig) plots. Alignment quality is evaluated by percentages of reads aligning to the expected transcribed regions (S11C Fig) and coverage metrics for known exon-exon junctions (S11D Fig). The observed patterns of splice site usage provide a general overview of alternative splicing patterns (S11E Fig). Finally, the expression of individual genes can be compared to the overall distribution to identify potentially up-regulated outliers (S11F Fig).

The preceding analysis was repeated in its entirety multiple times on standalone installations of the GMS with various hardware configurations on systems at our center, on consumer hardware available to ‘citizen scientists’, and on cloud computing services such as Amazon AWS EC2 (see S5 Table for examples). While potential alternative genome analysis platforms to the GMS are under development as both commercial and academic solutions, the breadth and comprehensiveness of cancer analysis described above and combination of additional features are to our knowledge unique to the GMS (S6 Table).

The GMS is a highly flexible and scalable system designed to enable genome analysts to maximize the yields from their data by increasing their ability to run a wide variety of analysis programs and explore the parameter space of each. The ability to reuse processing profiles offers reproducibility for complex processes (S12 and S13 Figs). A researcher can thus focus on just the variable of interest (e.g., tumor subtype, drug concentration, disease status, age of onset, etc.), confident that other variables (e.g., alignment software version, variant calling software parameters, reference genome sequence version, reference transcript annotation version, etc.) are truly constant. It also acts as the foundation for hypothesis testing of new computational methods. By allowing an analyst to produce alternatives to a given analysis pipeline with a few commands, the GMS permits an increased pace of tool and method development. Our testing of the GMS on cloud computing platforms demonstrates a mechanism for sharing complex results with collaborators or the community at large (S14 Fig). Finally, it allows standardization of analysis approaches when producing large sets of data in collaborative groups or consortia. A UML diagram of key GMS concepts is provided as S15 Fig.

In addition to the development advantages of the GMS described above, adoption of the GMS may provide practical advantages for a group attempting analysis of genome sequence data, especially in the context of cancer genomics. For example, a current adopter has access to well-vetted pipelines and tools for cancer genome analysis including: BWA, Strelka [46], VarScan2 [47], SomaticSniper [48], Pindel [49], GATK [50], BreakDancer [43], CREST, TIGRA_SV, ChimeraScan, the Tuxedo suite [51], the HTSeq and edgeR [52] combination, CopyCat (unpublished), and many more. Results include annotations according to cancer relevance; useful visualizations such as ‘lolliplot’ mutation diagrams, mutation spectrum diagrams, Circos [53] plots, XML session files for manual review in IGV, and intersection of altered genes with potential druggability from DGIdb.

## Availability and Future Directions

The HCC1395 analysis demonstrates the current abilities of the GMS to detect, summarize, visualize, and interpret the various types of somatic and germline events encountered in variant analysis such as SNVs, Indels, SVs, CNVs, differential expression, alternative expression and more. This analysis, while extensive, is still far from complete. Many further improvements are currently under way and will be released publicly at regular intervals. The HCC1395 data itself may also serve as a resource for external development. There are few publicly available datasets of this quality, with all three of the major sequence data types (WGS, exome, and RNA-seq), for a single tumor/normal pair, on a current platform, to facilitate development of tools. As the clinical sequencing analysis facilitated by the MedSeq pipeline is a primary area of interest, several new resources are under development for release in future versions of the GMS to further aid the interpretation of genomic events in a clinical translation and reporting context.

Flexibility, scalability, and ease of use have been the guiding principles behind development of the GMS. The GMS makes open, high-throughput genome analysis available to groups currently tasked to analyze the deluge of data from high-throughput sequencing experiments.

The GMS is made available under the open source GNU Lesser General Public License Version 3 (http://www.gnu.org/copyleft/lesser.html) and can be found on the GitHub Genome Institute pages (https://github.com/genome/gms).

## Supporting Information

S1 Fig GMS data management. (**A**) The top level command tree provides major entry points to data and tools in the system. (**B**) When developing extensions to the system, the bioinformatician’s sandbox is automatically recognized, and used instead of the production release.
(PDF)

S2 Fig Genome modeling tools. (**A**) The “genome tools” command tree is the primary way to access common bioinformatics components. An alias “gmt” is provided to make access less verbose. There are 134 top-level gmt sub-trees, with over 1,500 components available at the time of publication. This software is developed live on github and expands continually. (**B**) Each top-level command provides access to a list of tools, or further sub-trees. (**C**) The example “gmt fasta” sub-tree (highlighted below) contains script-like components for working with FASTA files. (**D**) Each tool has auto-generated help, built from the tool metadata. (**E**) The code for a GMT tool can be as simple as a short script. (**F**) Additional code can be added to the module to explicitly or dynamically generate other documentation.
(PDF)

S3 Fig CNV plot of HCC1395 tumor/normal comparison. (**A**) The top two panels show genome-wide ‘single-bam’ copy number plots for tumor and normal respectively. Extensive CNVs are apparent in the tumor as well as spurious peaks in both tumor and normal, especially around centromeres and telomeres. (**B**) The bottom panel shows a CNV plot of the difference in tumor versus normal for just chromosome 12 indicating a region of one, two, three and four copy gain with several known cancer genes affected including *KRAS*.
(PDF)

S4 Fig HCC1395 comparison of somatic SNV callers integrated in a single processing profile. (**A**) Variants called by three somatic SNV callers are summarized as a Venn diagram where the combination of calls from each combination of callers is indicated as a percentage of the total unique variants called. (**B**) The percentage of variant calls called by each combination of somatic variant callers that pass or fail manual review of read data in IGV are shown as a stacked bar plot.
(PDF)

S5 Fig Annotation of HCC1395 SNVs with respect to cancer relevant gene categories. (**A**) Genes with SNVs, insertions, or deletions in HCC1395 are displayed as a bar plot to show the number times the same amino acid mutation was observed in Cosmic. (**B**) The number of SNV mutated genes belonging to various cancer related gene categories are provided as a bar plot. A complete list of all somatic SNVs detected in HCC1395 is provided in S1 Data.
(PDF)

S6 Fig Visualization of amino acid position and Cosmic mutation recurrence data. Predicted amino acid effects are displayed as a ‘lollipop plot’ (aka mutation diagram) for mutations observed in HCC1395 and are contrasted to selected mutations from the Cosmic database for three example genes: (**A**) *BRCA2*, (**B**) *BCOR2*, and (**C**) *TP53*.
(PDF)

S7 Fig Indel plot of HCC1395 tumor/normal comparison. (**A**) A screenshot of an IGV session auto-generated by the GMS MedSeq pipeline is shown for a single deletion in *TAF1*. The source of sequence reads is indicated at the left of each panel. (**B**) The predicted amino acid effect of this deletion is shown as a mutation diagram with the mutation discovered in HCC1395 contrasted with mutations in this gene obtained from the Cosmic database and the position of protein domains indicated as colored bars.
(PDF)

S8 Fig HCC1395 data integration between WGS, exome and RNA-seq. Various statistics are summarized for exonic somatic SNV positions discovered by WGS and/or exome sequencing. (**A**) The distributions of normal sample read coverage (sequence depth) are shown as a histogram for WGS and exome data. (**B**) WGS read coverage is shown as a histogram for the tumor sample. (**C**) Tumor variant allele frequency (VAF) from WGS data is plotted against the VAF for exome data. (**D**) VAF from exome data is plotted against the VAF from RNA-seq data. The expression of level of each gene harboring an SNV is indicated on a colored scale (yellow indicates low expression; red indicates high expression).
(PDF)

S9 Fig HCC1395 SV/Fusion examples. (**A**) A list of putative ORF maintaining gene fusions detected with the SV pipeline using BreakDancer [43] and SquareDancer are provided as a bar plot indicating the number of supporting discordant read pairs. (**B**) A ‘pairoscope’ plot illustrates the supporting reads for one of these potential fusions between *PRTG* and *MALT1* on chromosomes 15 and 18. The complete list of predicted SVs from BreakDancer is provided as S4 Data.
(PDF)

S10 Fig Clonality plot for HCC1395. (**A**) A clonality plot, displaying the distribution of VAFs plotted against WGS sequencing coverage is provided as a kernel density plot. (**B**) To obtain a clonality plot that excludes regions of copy number alteration, VAFs were limited to those from selected regions of a chromosome 21 with a copy-neutral state. These regions are indicated as dotted boxes on a plot of chromosome positions against tumor-normal copy number difference, where a value of 0 represents no difference in copy number between tumor and normal.
(PDF)

S11 Fig HCC1395 RNA expression and splicing. A sample of graphs automatically generated by the GMS to interpret RNA-seq results. (**A**) Library quality assessed by observed insert size distribution. (**B**) End bias plots showing the distribution of RNA-seq reads across the length of sequenced transcripts. (**C**) Percentage of reads aligning to the expected transcribed and non-transcribed regions. (**D**) Sequence coverage of known exon-exon junctions. (**E**) The observed patterns of splice site usage (**F**) The expression of an individual gene, *NPM1*, compared to the overall distribution of gene expression values.
(PDF)

S12 Fig Cohort analysis. Both the concept of “subject” and “model” can be applied at multiple levels of granularity. This example builds on Fig 2B, wherein several individual subjects are modeled individually, using a processing profile that aims to analyze a single sample in a consistent fashion. Following that, a model of a different type might be defined that draws further conclusions about a cohort, given the prior conclusions of its input models. In this example a mutational significance model runs the MuSiC suite, identifying significantly mutated genes in the cohort.
(PDF)

S13 Fig Build view. The “genome model build view” command displays the status of all of the tasks within a build workflow. The following images show the build process for the WGS somatic variation build used in the example analysis. This is the same workflow illustrated in Fig 3. Image (**A**) shows the header for the build report, including the name of the model, the user who launched the build, and the ID for the processing profile. A table of steps is then presented. Each step has a database identifier, and also an ID for the job in the cluster management system (LSF). The status of the job is indicated in color. Where steps are nested, indentation of the name is used to suggest the situation visually. Variant detectors such as Pindel (**A**) and Breakdancer (**B**) have subordinate workflows, dividing work by genomic region. The TIGRA in silico SV validation step also divides work by chromosome (**B**, **C** and **D**), and is performed for each SV detection approach. For this build, the execution of VarScan2 and Strelka “shortcut” (**B**), indicating that the data set required already exists for the same inputs and parameters, presumably because of a prior build performing work with some overlap. The end of the report shows steps that merge results across approaches, and perform final annotation of variants.
(PDF)

S14 Fig Web interface. The GMS web search interface provides high-speed access to large volumes of data. (**A**) It offers separate tabs to allow searching by model, build, processing profiles, instrument data, or subject. The free-form search box provides direct access to querying the database without the analyst knowing exact field names and nomenclature. The results in each tab have links to the other related entities in the system, as well as the ability to drill down for additional detail about the entity in question. This example shows a search for models related to the HCC1395 cell line subject. (**B**) This page for an individual sample shows general data about the sample, followed by a link to information about DNA fragment libraries, behind which are specifics about instrument data. Below this page begins a list of models that have been made with this sample as the subject. (**C**) Each listed model shows its processing profile and inputs, as well as a list of build attempts, and respective build statuses. In this example, exome-capture based alignment and variant detection are running. The genotype microarray analysis of the same sample has completed successfully, but prior to that had one failed attempt at processing. (**D**) The fourth image shows details for a specific build, including a list of specific steps, and the status of each on the compute cluster. Links are present to the log files of each step, and also to the log file for the build process as a whole.
(PDF)

S15 Fig UML diagram of key GMS components. A unified modeling language (UML) diagram of some critical components of the GMS.
(PDF)

S1 Table HCC1395/BL whole genome (DNA) sequence metrics. (PDF)

S2 Table HCC1395/BL whole exome (DNA) sequence metrics. (PDF)

S3 Table HCC1395/BL Transcriptome (RNA) sequence metrics. (PDF)

S4 Table HCC1395/BL selected candidate cancer associated SNVs. (PDF)

S5 Table Test hardware configurations. (PDF)

S6 Table The GMS, conceptually related resources, and their features. (PDF)

S1 Data HCC1395/BL tier 1 somatic SNVs (Sniper, VarScan, Strelka). ‘Top’ transcript variant annotations of SNVs from the somatic variation pipeline, cancer annotations, Cosmic annotations and expression status from the MedSeq pipeline.
(ZIP)

S2 Data HCC1395/BL tier 1 somatic Indels (GATK, VarScan, Pindel, Strelka). ‘Top’ transcript variant annotations of Indels from the somatic variation pipeline, cancer annotations and Cosmic annotations from the MedSeq pipeline.
(ZIP)

S3 Data HCC1395/BL somatic CNVs (cnvhmm). CNV segments and CNV amplified and deleted genes from the MedSeq pipeline.
(ZIP)

S4 Data HCC1395/BL somatic SVs (breakdancer). Annotated SV predictions from the somatic variation pipeline and candidate SV fusions from the MedSeq pipeline.
(ZIP)

S5 Data HCC1395/BL RNA expression values (Cufflinks). Gene and transcript expression values (FPKMs) from the MedSeq pipeline.
(ZIP)

S6 Data HCC1395/BL observed splice junctions and their abundance (Tophat). Exon-exon junctions identified as expressed and annotated in the RNA-seq pipeline.
(ZIP)

S7 Data HCC1395/BL RNA gene fusions (ChimeraScan). RNA fusions predicted by ChimeraScan in the RNA-seq pipeline.
(ZIP)

S1 Text Supplementary Methods and References. (PDF)
