# Supplementary material for: Genome Modeling System: A Knowledge Management Platform for Genomics
Source: PLoS Comput Biol. 2015 Jul 9;11(7):e1004274. doi: 10.1371/journal.pcbi.1004274 (PMC4497734; doi:10.1371/journal.pcbi.1004274)
Supplement: S1 Fig — (A) The top level command tree provides major entry points to data and tools in the system. (B) When developing extensions to the system, the bioinformatician’s sandbox is automatically recognized, and used instead of the production release. (PDF) [file pcbi.1004274.s001.pdf]

A. The top-level command tree of 'genome'

ss — bash — 86×34

```
ssmith@blade12-1-1 > genome
Sub-commands for genome:
 db                    ...  external database interfaces
 disk                  ...  work with allocations, volumes, etc
 feature-list          ...  work with feature-lists
 individual            ...  work with individuals
 instrument-data       ...  work with instrument data
 library               ...  work with libraries
 model                 ...  work with models
 model-group           ...  work with model-groups
 population-group      ...  work with population groups
 processing-profile    ...  work with processing profiles.
 project               ...  work with projects
 project-part          ...  work with project parts
 report                     work with reports
 sample                ...  work with samples
 software-result       ...  work with software results
 subject               ...  work with subjects
 sys                   ...  work with OS integration
 task                  ...  work with tasks
 taxon                 ...  work with taxons
 tools                 ...  bioinformatics tools for genomics
```

B. Modifications of the genome source code are automatically recognized and used

mgriffit — ssh — 86×34

ssh

```
vagrant@precise64 /opt/gms/TML8B20/sw/genome/lib/perl/Genome (gms-pub)> genome
Using libraries at /opt/gms/TML8B20/sw/genome/lib/perl
Sub-commands for genome:
 analysis-project      ...  work with analysis projects
 config                ...  commands that deal with analysis project configuration
 db                    ...  external database interfaces
 disk                  ...  commands that work with allocations, volumes, etc
```
