# Supplementary material for: Genome Modeling System: A Knowledge Management Platform for Genomics
Source: PLoS Comput Biol. 2015 Jul 9;11(7):e1004274. doi: 10.1371/journal.pcbi.1004274 (PMC4497734; doi:10.1371/journal.pcbi.1004274)
Supplement: S2 Fig — (A) The “genome tools” command tree is the primary way to access common bioinformatics components. An alias “gmt” is provided to make access less verbose. There are 134 top-level gmt sub-trees, with over 1,500 components available at the time of publication. This software is developed live on github and expands continually. (B) Each top-level command provides access to a list of tools, or further sub-trees. (C) The example “gmt fasta” sub-tree (highlighted below) contains script-like components for working with FASTA files. (D) Each tool has auto-generated help, built from the tool metadata. (E) The code for a GMT tool can be as simple as a short script. (F) Additional code can be added to the module to explicitly or dynamically generate other documentation. (PDF) [file pcbi.1004274.s002.pdf]

A. The ‘genome model tools’ (gmt) command

```
ssmith — ssmith@blade13-4-4: ~ — ssh — 118×53
ssmith@blade13-4-4: ~        0:1.0 bash        ssh ...

ssmith@blade13-4-10 ~> gmt
Sub-commands for gmt:
 smalt                          Tools to run smalt or work with its output files.
 tophat                         Tools to run Tophat or work with its output files.
 ssaha2                         Tools to run SSAHA2 or work with its output files.
 bfast                          Tools to run Bfast or work with its output files.
 tigra-sv                       Tools to run tigra_sv or work with its output files.
 blat                           Tools to run Blat or work with its output files.
 bwa                            Tools to run BWA or work with its output files.
 crossmatch                     Tools to run Crossmatch or work with its output files.
 picard                         Tools to run the Java toolkit Picard and work with SAM/BAM
                                 format files.
 novocraft                      Tools to run novocraft or work with its output files.
 bsmap                          Tools to run BSMAP or work with its output files.
 mosaik                         Tools to run Mosaik or work with its output files.
```

B. Each top-level command provides access to a list of tools, or further sub-trees

```
 bowtie                             tools to work with the Bowtie aliger
 breakdancer                        discovers structural variation using breakdancer
 bwa-sw                             tools to work with Ssaha output
 chimera-slayer                     Tool to run chimera detector: chimera_slayer
 complete-genomics              ... base class for commands which delegate to sub-commands
 copy-cat                       ... the CopyCat copy number analysis tools
 cufflinks                          Tools to run Cufflinks or work with its output files.
 detect-variants                    A selection of variant detectors.
 dgidb                          ... Toolkit for DGIDB related process
 ensembl                        ... Tools to work with the local Ensembl API.
 epitope-prediction             ... Different pipeline steps for Immune Epitope Prediction for
 far                            ... To trim adaptor sequences
 fasta                              Tools for working with FASTA and Qual files
 fastq                              tools for working with FASTQ files
 fastqc                             Tools to run the Java toolkit FastQC and work with the
                                     output reports.
 fastx                              Tools to run Fastx or work with its output files.
 galaxy                         ... the Galaxy web interface
 gatk                               tools to work with Gatk output
 gene-torrent                       no description!!!: define 'doc' in the class definition for
                                     Genome::Model::Tools::GeneTorrent
 gtf                            ... Tools to work with gtf format annotation files.
 htseq                          ... htseq tools (htseq-count and htseq-qa) work with
                                     gene/transcript hit-counts
 lift-over                          wrapper for the UCSC liftOver tool with support for
                                     additional input formats, maintaining additional columns
```

C. The ‘gmt fasta’ sub-tree contains script-like components for working with FASTA files

```
ss — bash — 116×45
ssmith@blade12-1-1 > gmt fasta
Sub-commands for genome tools fasta:
 apply-diff            --diff=? --input=...  applies seq inserts and deletes from a diff file to a fasta
                                              file
 chunk                 --chunk-size=? --...  Divide fasta into chunk by chunk_size
 concat                --input-files=?[,...  Mixin that gives commands color option
 deduplicator          --fasta-file=? [-...  remove duplicates from a file of reads
 diff                  [--debug] FILE1 F...  use KDiff3 to show differences between fasta data files
 dust                  --dusted-file=? -...  Tools for working with FASTA and Qual files
 filter-ids            [--verbose] [--wh...  filter sequences from a fasta file based on patterns
                                              applied to the IDs
 orient                --fasta-file=? [-...  Orients FASTA (and Quality) files by blastn given sense and
                                              anti-sense sequences
 remove-n              --fasta-file=? [-...  remove reads from file containing N
 sanitize              --fasta-file=? [-...  Cleans FASTA (and Quality) files
 screen-vector         --fasta-file=? [-...  (Fnq = Fasta And Quality) screen for vector
 sliding-windows       --fasta-file=? --...  Tools for working with FASTA and Qual files
 sort-by-name          --input-fasta=? [...  Sorts a fasta by sequence name
```

D. Each tool has auto-generated help

```
ssmith — ssmith@linus43: ~ — ssh — 100×40
ssmith@linus43: ~                         bash

ssmith@linus43 ~> gmt fasta filter-ids -h

USAGE
 gmt fasta filter-ids [--verbose] [--whitelist-regex=?] [--blacklist-regex=?]
    INPUT-FILENAME OUTPUT-FILENAME

SYNOPSIS
    gmt fasta filter-ids in.fa out.fa --whitelist '^(\d+|X,Y)$' --blacklist '6'

REQUIRED INPUTS
  INPUT-FILENAME
    the input file
  OUTPUT-FILENAME
    the path to the file that will be created

OPTIONAL INPUTS
  whitelist-regex
    include only IDs that match this pattern
  blacklist-regex
    exclude any IDs that match this pattern

OPTIONAL PARAMS
  verbose
    more messages

DESCRIPTION
    This tool filters a FASTA sequence file, removing entries based on the ID in the FASTA header.
    If the "whitelist regex" (-w) option is supplied, only IDs that match this regular expression
    will be included. If the "blacklist regex" (-b) option is supplied, only IDs that do NOT match
    this regular expression will be included.


NOTE

    If an ID matches both the black list and the white list, it is skipped.


ssmith@linus43 ~>
```

E. The code for a GMT tool can be as simple as a short script

```perl
package Genome::Model::Tools::Fasta::FilterIds;
use strict;
use warnings;
use Genome;
use Bio::SeqIO;

class Genome::Model::Tools::Fasta::FilterIds {
    is => 'Command::V2',
    has_input => [
        input_filename => {
            is => 'FilesystemPath', shell_args_position => 1,
            doc => 'the input file',
        },
        output_filename =>  {
            is => 'FilesystemPath', shell_args_position => 2,
            doc => 'the path to the file that will be created',
        },
        whitelist_regex => {
            is => 'Text', is_optional => 1,
            doc => 'include only IDs that match this pattern',
        },
        blacklist_regex => {
            is => 'Text', is_optional => 1,
            doc => 'exclude any IDs that match this pattern',
        },
    ],
    has_param => [
        verbose => {
            is => 'Boolean', is_optional => 1,
            doc => 'more messages'
        },
    ],
    doc => "filter sequences from a fasta file based on patterns applied to the IDs",
};

sub execute {
    my $self = shift;
    my $input_filename = $self->input_filename;
    my $output_filename = $self->output_filename;
    my $verbose = $self->verbose;

    my $blacklist_regex = $self->blacklist_regex;
    my $whitelist_regex = $self->whitelist_regex;

    my $reader = Bio::SeqIO->new( '-file' => '< '.$input_filename, '-format' => 'fasta');
    my $writer = Bio::SeqIO->new( '-file' => '> '.$output_filename, '-format' => 'fasta');

    while (my $seq = $reader->next_seq) {
        my $id = $seq->id;
        if ($blacklist_regex and $id =~ $blacklist_regex) {
            $self->status_message("skipping $id because it matches the blacklist pattern");
            next;
        }
        elsif ($whitelist_regex and not $id =~ $whitelist_regex) {
            $self->status_message("skipping $id because it does not match the whitelist pattern");
            next;
        }
        elsif ($verbose) {
            $self->status_message("keeping $id");
        }
        $writer->write_seq($seq);
    }
    return 1;
}
```

F. Additional code can be added to the module to explicitly or dynamically generate other documentation

```perl

sub help_synopsis {
return <<'EOS'
    gmt fasta filter-ids in.fa out.fa --whitelist '^(\d+|X,Y)$' --blacklist '6'
EOS
}

sub help_detail {
    return <<EOS
This tool filters a FASTA sequence file, removing entries based on the ID in the FASTA header.
If the "whitelist regex" (-w) option is supplied, only IDs that match this regular expression will be included.
If the "blacklist regex" (-b) option is supplied, only IDs that do NOT match this regular expression will be included.
EOS
}

sub _additional_help_sections {
    return (
        "NOTE" =>
        "If an ID matches both the black list and the white list, it is skipped."
  );
}

sub _doc_manual_body {
    # expect to return POD
    my $help = shift->help_detail;
    $help =~ s/\n+$/\n/g;
    return $help;
}

sub _doc_authors {
    return <<EOS
 Scott Smith
 Edward Belter
EOS
}

sub _doc_copyright_years { (2013) }

sub _doc_license {
    my $self = shift;
    my (@y) = $self->_doc_copyright_years;
    my $range;
    if (@y == 1) { $range = "$y[0]"; }
    elsif (@y > 1) { $range = "$y[0]-$y[-1]"; }
    return <<EOS
Copyright (C) $range Washington University in St. Louis.

It is released under the Lesser GNU Public License (LGPL) version 3.  See the
associated LICENSE file in this distribution.
EOS
}

sub _doc_credits {
    return ('','This software was created with funding fromthe National Human Genome Research Institute.');
}

sub _doc_see_also {
    return <<EOS
B<Genome>(3)
EOS
}

1;
```
