# Supplementary material for: Genome Modeling System: A Knowledge Management Platform for Genomics
Source: PLoS Comput Biol. 2015 Jul 9;11(7):e1004274. doi: 10.1371/journal.pcbi.1004274 (PMC4497734; doi:10.1371/journal.pcbi.1004274)
Supplement: S3 Fig — (A) The top two panels show genome-wide ‘single-bam’ copy number plots for tumor and normal respectively. Extensive CNVs are apparent in the tumor as well as spurious peaks in both tumor and normal, especially around centromeres and telomeres. (B) The bottom panel shows a CNV plot of the difference in tumor versus normal for just chromosome 12 indicating a region of one, two, three and four copy gain with several known cancer genes affected including KRAS. (PDF) [file pcbi.1004274.s003.pdf]

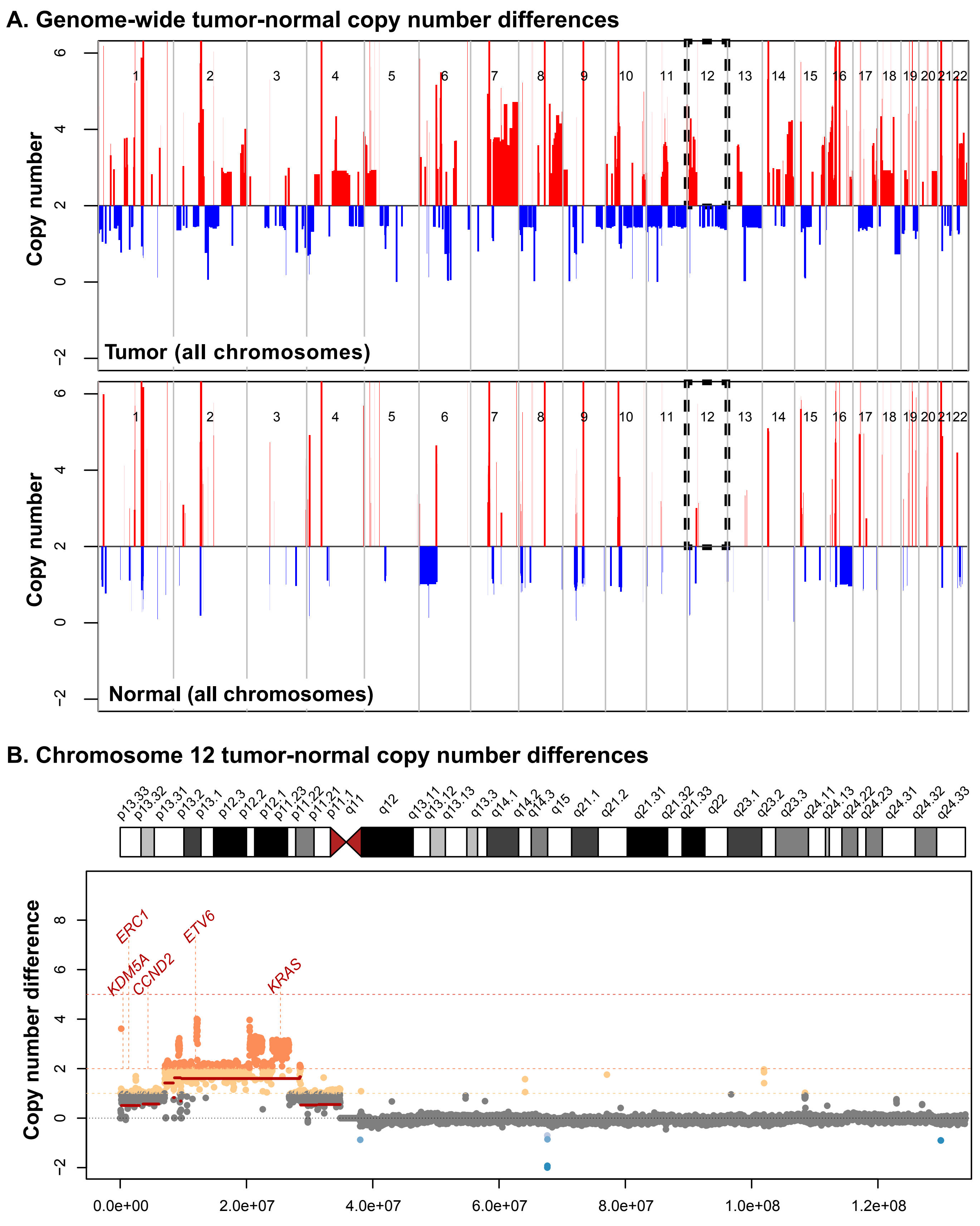
A. Genome-wide tumor-normal copy number differences
Copy number
Tumor (all chromosomes)
Normal (all chromosomes)
1
2
3
4
5
6
7
8
9
10
11
12
13
14
15
16
17
18
19
20
21
22
B. Chromosome 12 tumor-normal copy number differences
Copy number difference
ERC1
KDM5A
CCND2
ETV6
KRAS
p13.33
p13.32
p13.31
p13.2
p13.1
p12.3
p12.2
p12.1
p11.23
p11.22
p11.21
p11.1
q11
q12
q13.11
q13.12
q13.13
q13.3
q14.1
q14.2
q14.3
q15
q21.1
q21.2
q21.31
q21.32
q21.33
q22
q23.1
q23.2
q23.3
q24.11
q24.13
q24.22
q24.23
q24.31
q24.32
q24.33
0.0e+00
2.0e+07
4.0e+07
6.0e+07
8.0e+07
1.0e+08
1.2e+08
