# Supplementary figures and images for: Genome Modeling System: A Knowledge Management Platform for Genomics
Source: PLoS Comput Biol. 2015 Jul 9;11(7):e1004274. doi: 10.1371/journal.pcbi.1004274 (PMC4497734; doi:10.1371/journal.pcbi.1004274)

A. Variant caller overlap for all SNVs

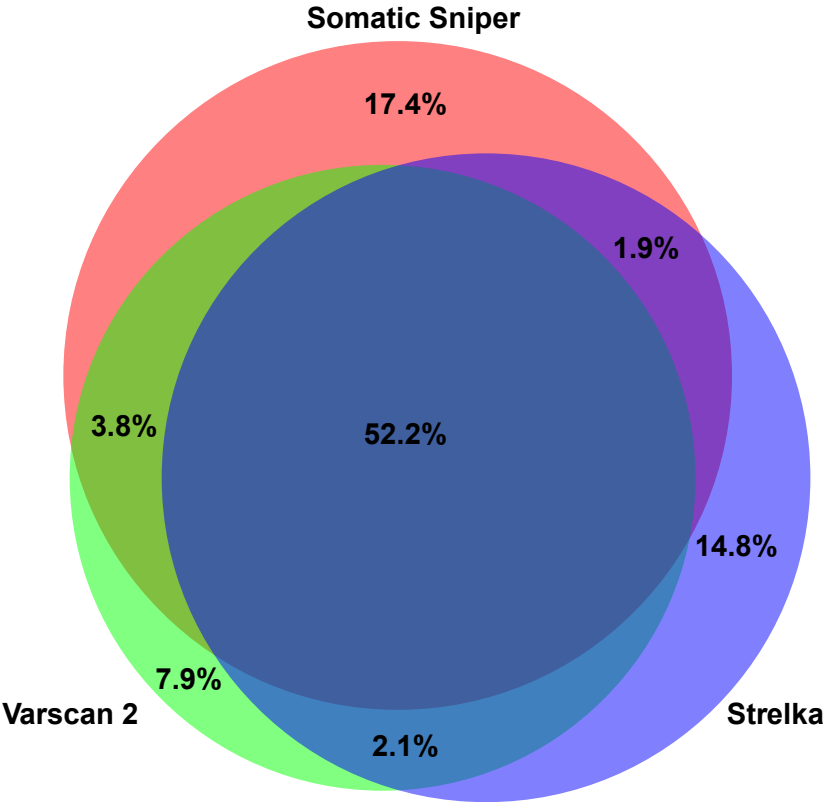

B. Percentage of variants passing manual review

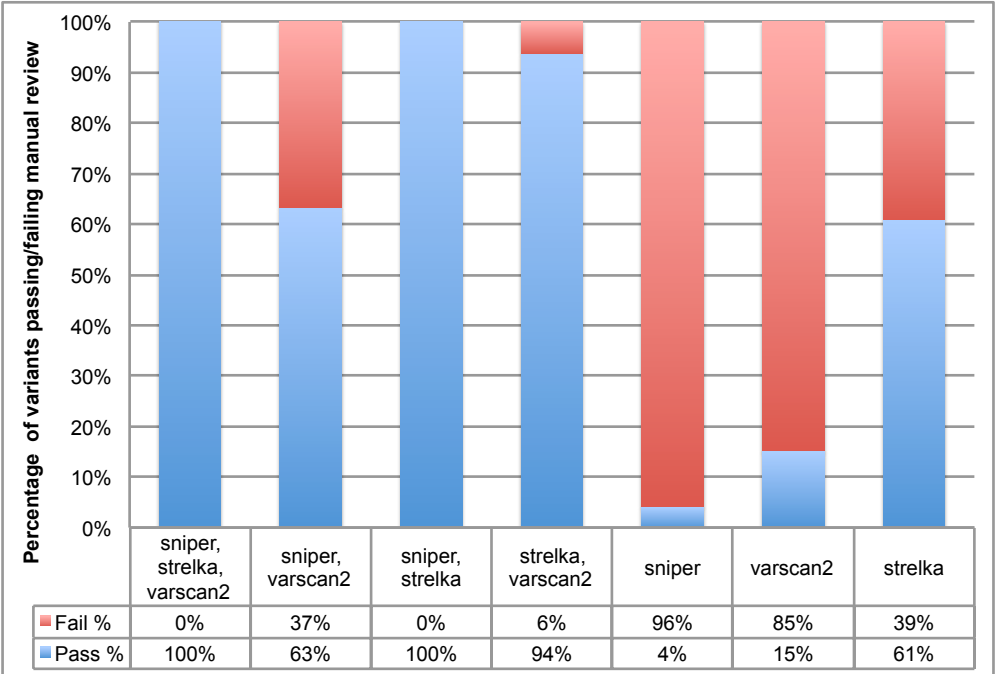

Supplement: S4 Fig — (A) Variants called by three somatic SNV callers are summarized as a Venn diagram where the combination of calls from each combination of callers is indicated as a percentage of the total unique variants called. (B) The percentage of variant calls called by each combination of somatic variant callers that pass or fail manual review of read data in IGV are shown as a stacked bar plot. (PDF) [file pcbi.1004274.s004.pdf]

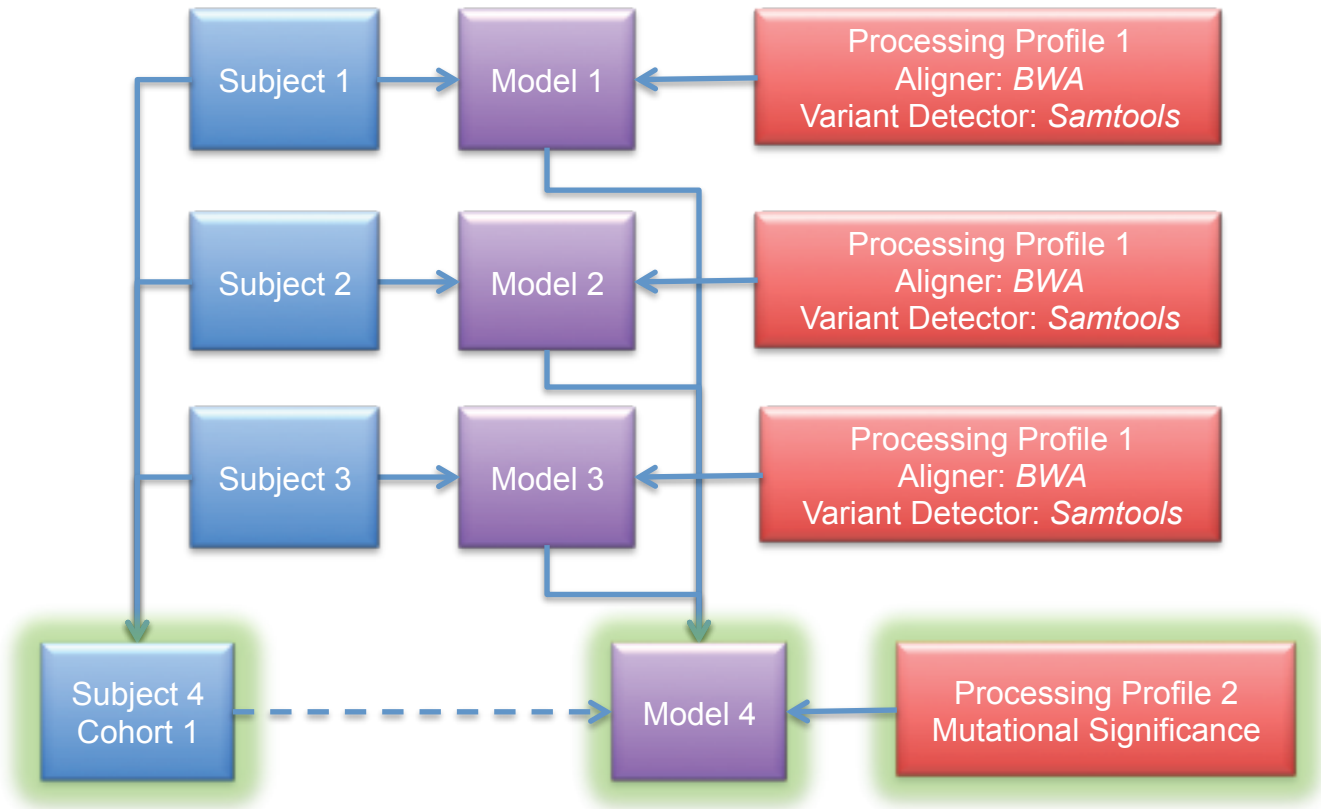

Supplement: S12 Fig — Both the concept of “subject” and “model” can be applied at multiple levels of granularity. This example builds on Fig 2B, wherein several individual subjects are modeled individually, using a processing profile that aims to analyze a single sample in a consistent fashion. Following that, a model of a different type might be defined that draws further conclusions about a cohort, given the prior conclusions of its input models. In this example a mutational significance model runs the MuSiC suite, identifying significantly mutated genes in the cohort. (PDF) [file pcbi.1004274.s012.pdf]

# Genome Modeling System

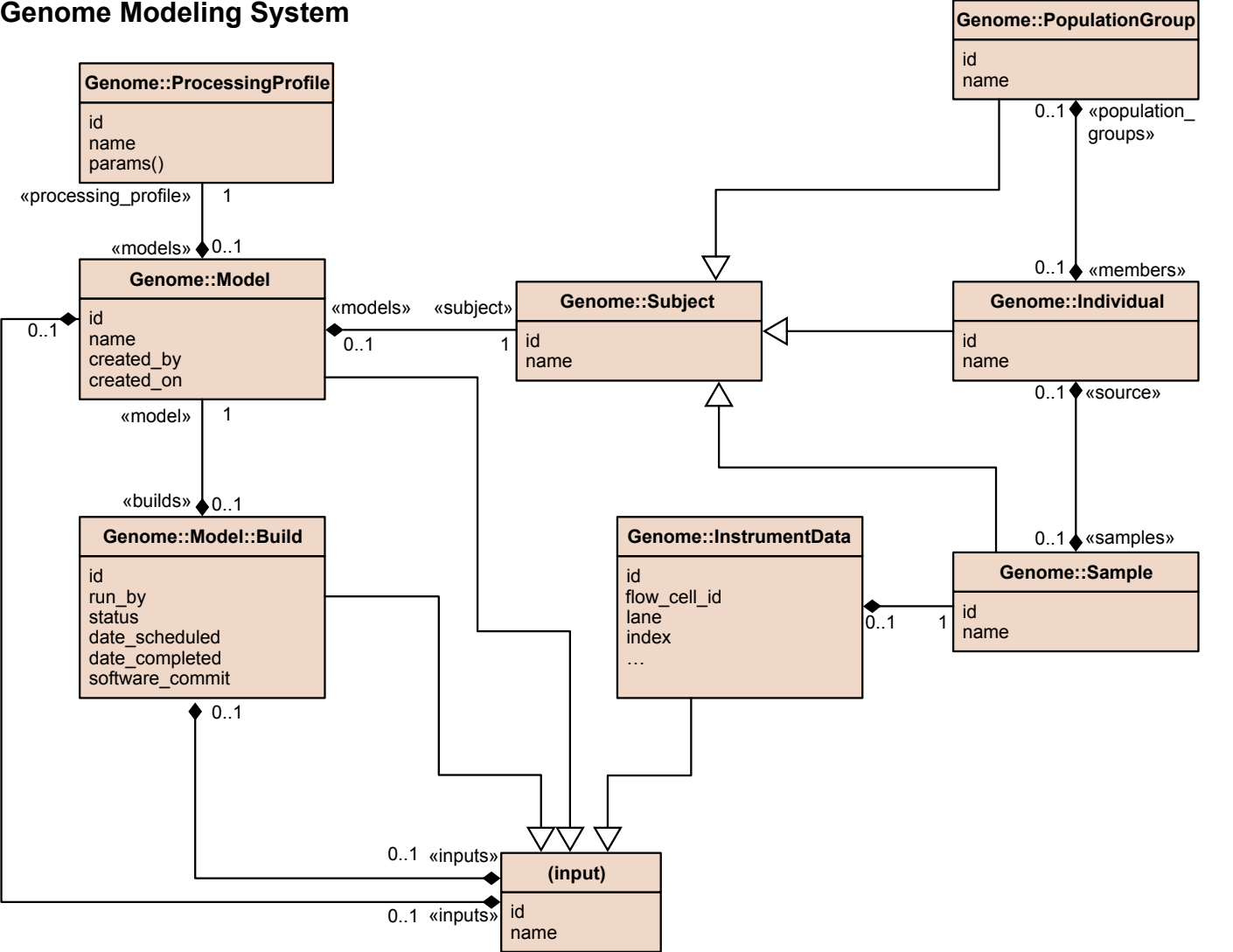

Supplement: S15 Fig — A unified modeling language (UML) diagram of some critical components of the GMS. (PDF) [file pcbi.1004274.s015.pdf]
