# Supplementary material for: Genome Modeling System: A Knowledge Management Platform for Genomics
Source: PLoS Comput Biol. 2015 Jul 9;11(7):e1004274. doi: 10.1371/journal.pcbi.1004274 (PMC4497734; doi:10.1371/journal.pcbi.1004274)

A. HCC1395 mutations previously observed in cancer according to Cosmic

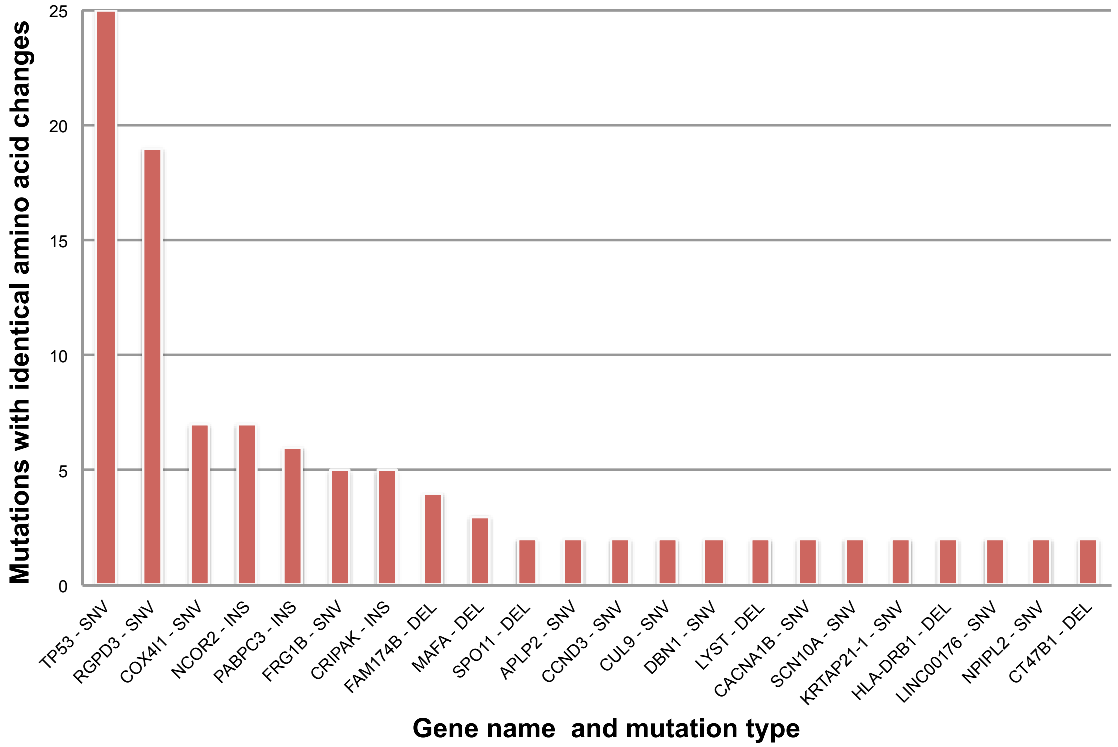

B. HCC1395 mutated genes grouped by cancer annotations

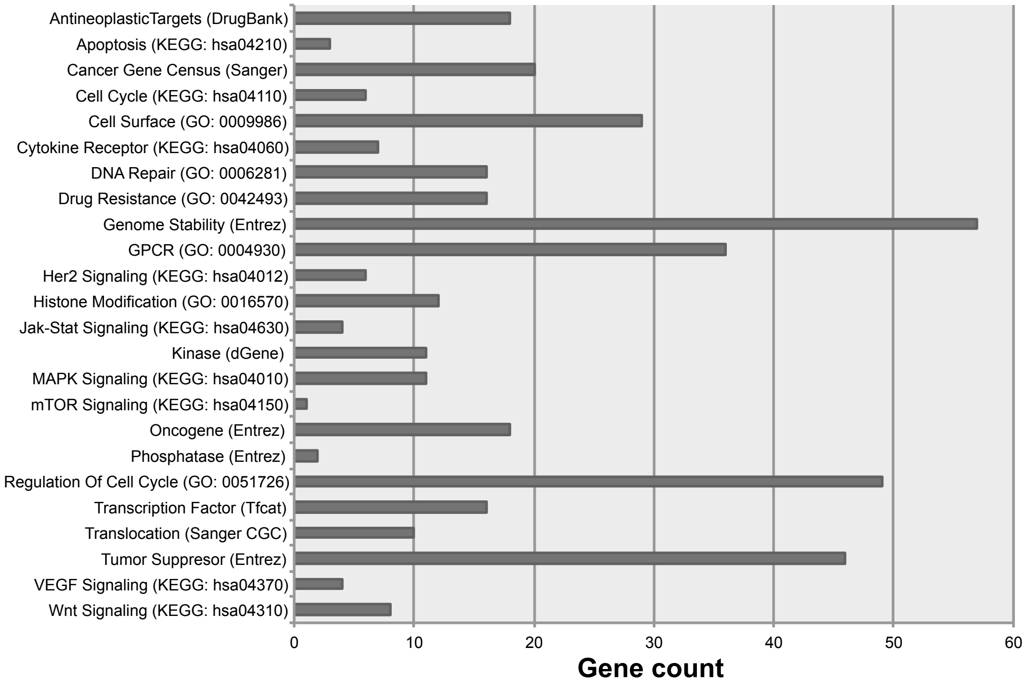

Supplement: S5 Fig — (A) Genes with SNVs, insertions, or deletions in HCC1395 are displayed as a bar plot to show the number times the same amino acid mutation was observed in Cosmic. (B) The number of SNV mutated genes belonging to various cancer related gene categories are provided as a bar plot. A complete list of all somatic SNVs detected in HCC1395 is provided in S1 Data. (PDF) [file pcbi.1004274.s005.pdf]
