# Supplementary material for: Genome Modeling System: A Knowledge Management Platform for Genomics
Source: PLoS Comput Biol. 2015 Jul 9;11(7):e1004274. doi: 10.1371/journal.pcbi.1004274 (PMC4497734; doi:10.1371/journal.pcbi.1004274)
Supplement: S6 Fig — Predicted amino acid effects are displayed as a ‘lollipop plot’ (aka mutation diagram) for mutations observed in HCC1395 and are contrasted to selected mutations from the Cosmic database for three example genes: (A) BRCA2, (B) BCOR2, and (C) TP53. (PDF) [file pcbi.1004274.s006.pdf]

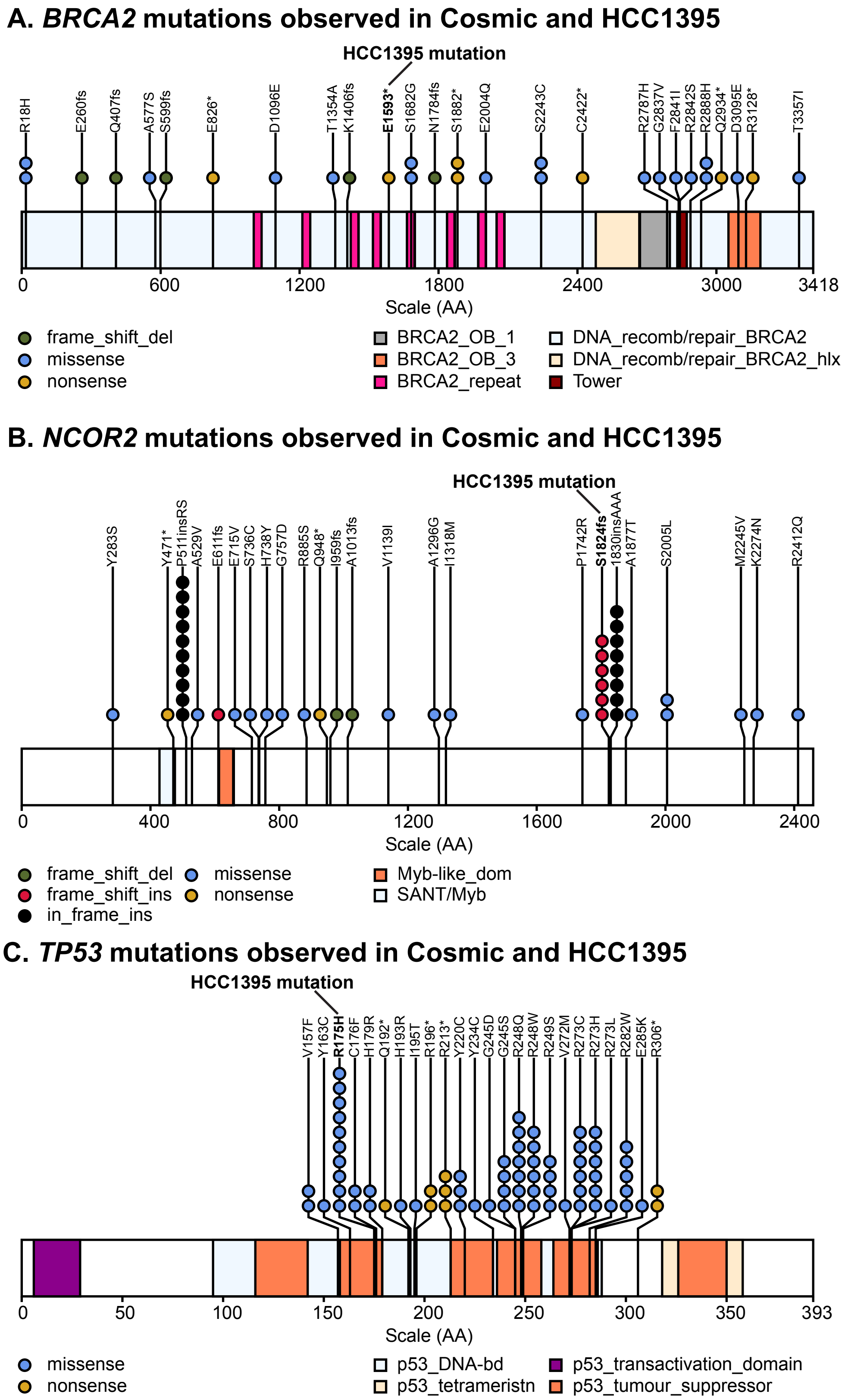
A. BRCA2 mutations observed in Cosmic and HCC1395
HCC1395 mutation
R18H
E260fs
Q407fs
A577S
S599fs
E826*
D1096E
T1354A
K1406fs
E1593*
S1682G
N1784fs
S1882*
E2004Q
S2243C
C2422*
R2787H
G2837V
F2841I
R2842S
R2888H
Q2934*
D3095E
R3128*
T3357I
0
600
1200
1800
2400
3000
3418
Scale (AA)
frame_shift_del
missense
nonsense
BRCA2_OB_1
BRCA2_OB_3
BRCA2_repeat
DNA_recomb/repair_BRCA2
DNA_recomb/repair_BRCA2_hlx
Tower
B. NCOR2 mutations observed in Cosmic and HCC1395
HCC1395 mutation
Y283S
Y471*
P511insRS
A529V
E611fs
E715V
S736C
H738Y
G757D
R885S
Q948*
I959fs
A1013fs
V1139I
A1296G
I1318M
P1742R
S1824fs
1830insAAA
A1877T
S2005L
M2245V
K2274N
R2412Q
0
400
800
1200
1600
2000
2400
Scale (AA)
frame_shift_del
frame_shift_ins
in_frame_ins
missense
nonsense
Myb-like_dom
SANT/Myb
C. TP53 mutations observed in Cosmic and HCC1395
HCC1395 mutation
V157F
Y163C
R175H
C176F
H179R
Q192*
H193R
I195T
R196*
R213*
Y220C
Y234C
G245D
G245S
R248Q
R248W
R249S
V272M
R273C
R273H
R273L
R282W
E285K
R306*
0
50
100
150
200
250
300
350
393
Scale (AA)
missense
nonsense
p53_DNA-bd
p53_tetrameristn
p53_transactivation_domain
p53_tumour_suppressor
