# Supplementary material for: Genome Modeling System: A Knowledge Management Platform for Genomics
Source: PLoS Comput Biol. 2015 Jul 9;11(7):e1004274. doi: 10.1371/journal.pcbi.1004274 (PMC4497734; doi:10.1371/journal.pcbi.1004274)

A. Automated IGV session creation facilitates manual review of Indels

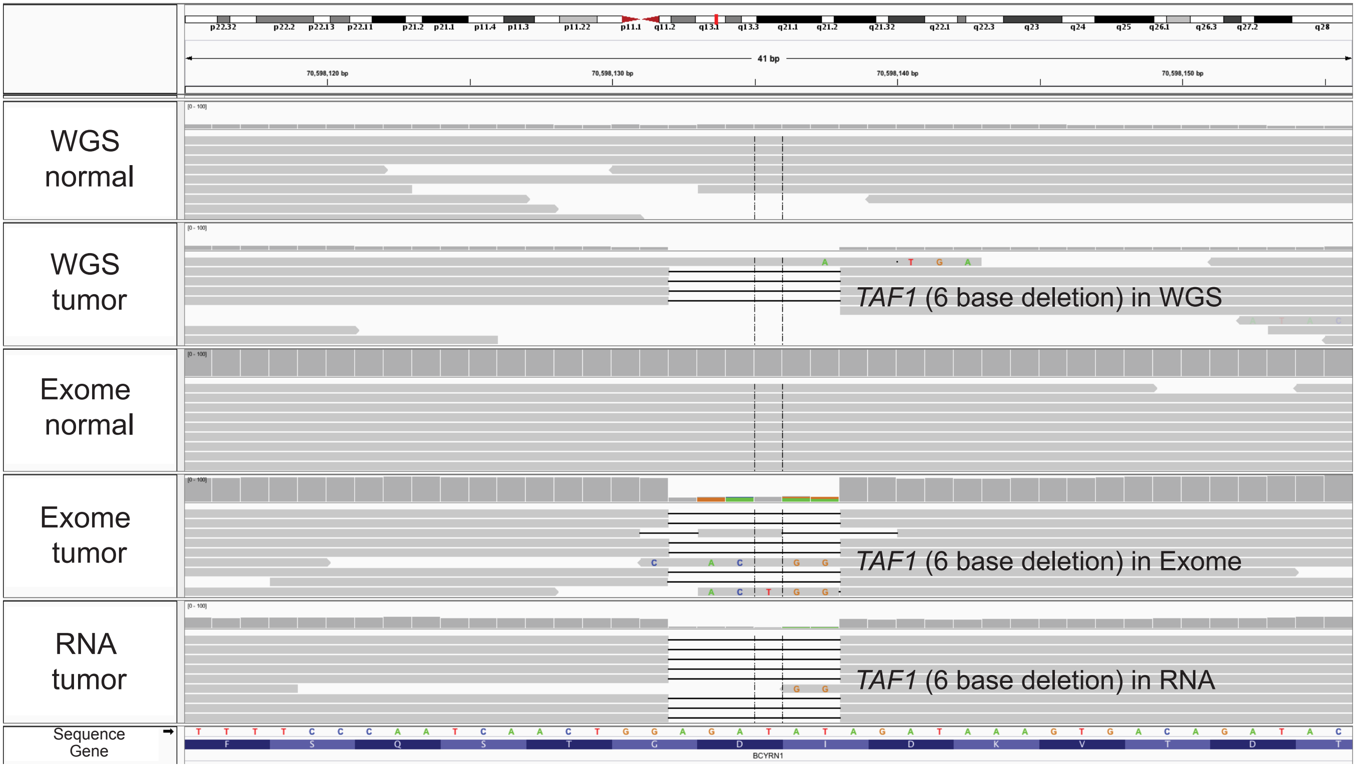

B. *TAF1* mutations. Amino acid and protein domain context

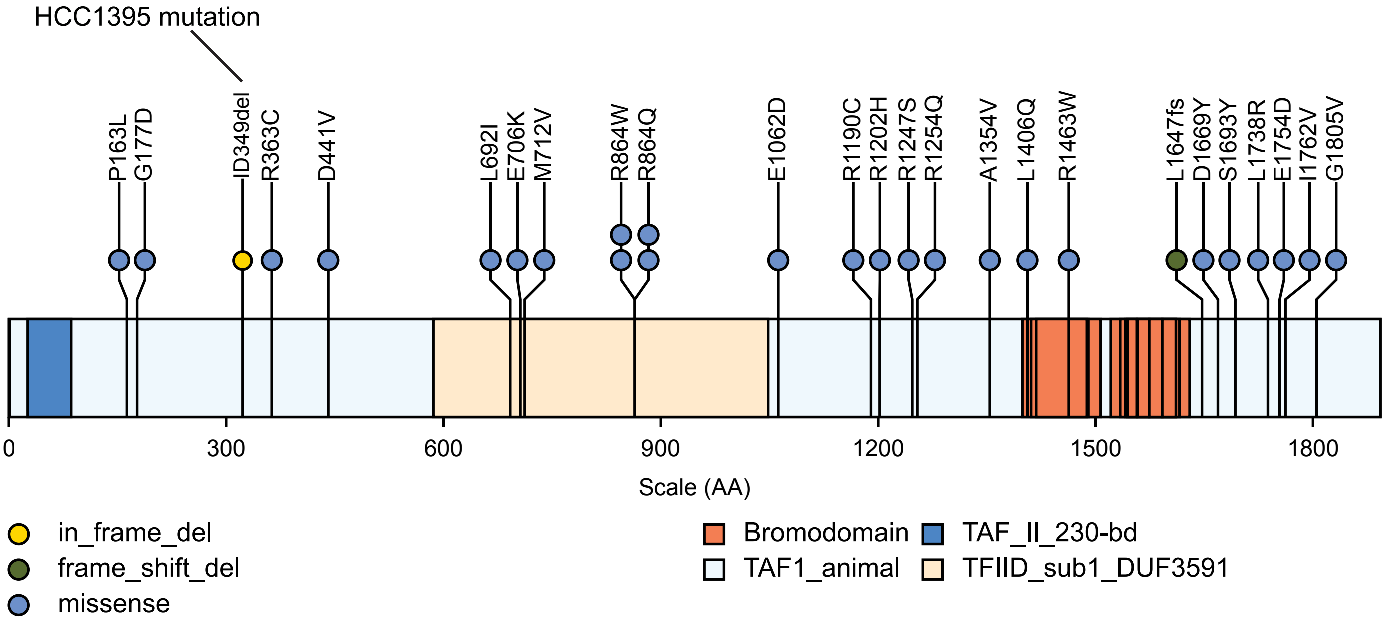

Supplement: S7 Fig — (A) A screenshot of an IGV session auto-generated by the GMS MedSeq pipeline is shown for a single deletion in TAF1. The source of sequence reads is indicated at the left of each panel. (B) The predicted amino acid effect of this deletion is shown as a mutation diagram with the mutation discovered in HCC1395 contrasted with mutations in this gene obtained from the Cosmic database and the position of protein domains indicated as colored bars. (PDF) [file pcbi.1004274.s007.pdf]
