# Supplementary material for: Genome Modeling System: A Knowledge Management Platform for Genomics
Source: PLoS Comput Biol. 2015 Jul 9;11(7):e1004274. doi: 10.1371/journal.pcbi.1004274 (PMC4497734; doi:10.1371/journal.pcbi.1004274)
Supplement: S8 Fig — Various statistics are summarized for exonic somatic SNV positions discovered by WGS and/or exome sequencing. (A) The distributions of normal sample read coverage (sequence depth) are shown as a histogram for WGS and exome data. (B) WGS read coverage is shown as a histogram for the tumor sample. (C) Tumor variant allele frequency (VAF) from WGS data is plotted against the VAF for exome data. (D) VAF from exome data is plotted against the VAF from RNA-seq data. The expression of level of each gene harboring an SNV is indicated on a colored scale (yellow indicates low expression; red indicates high expression). (PDF) [file pcbi.1004274.s008.pdf]

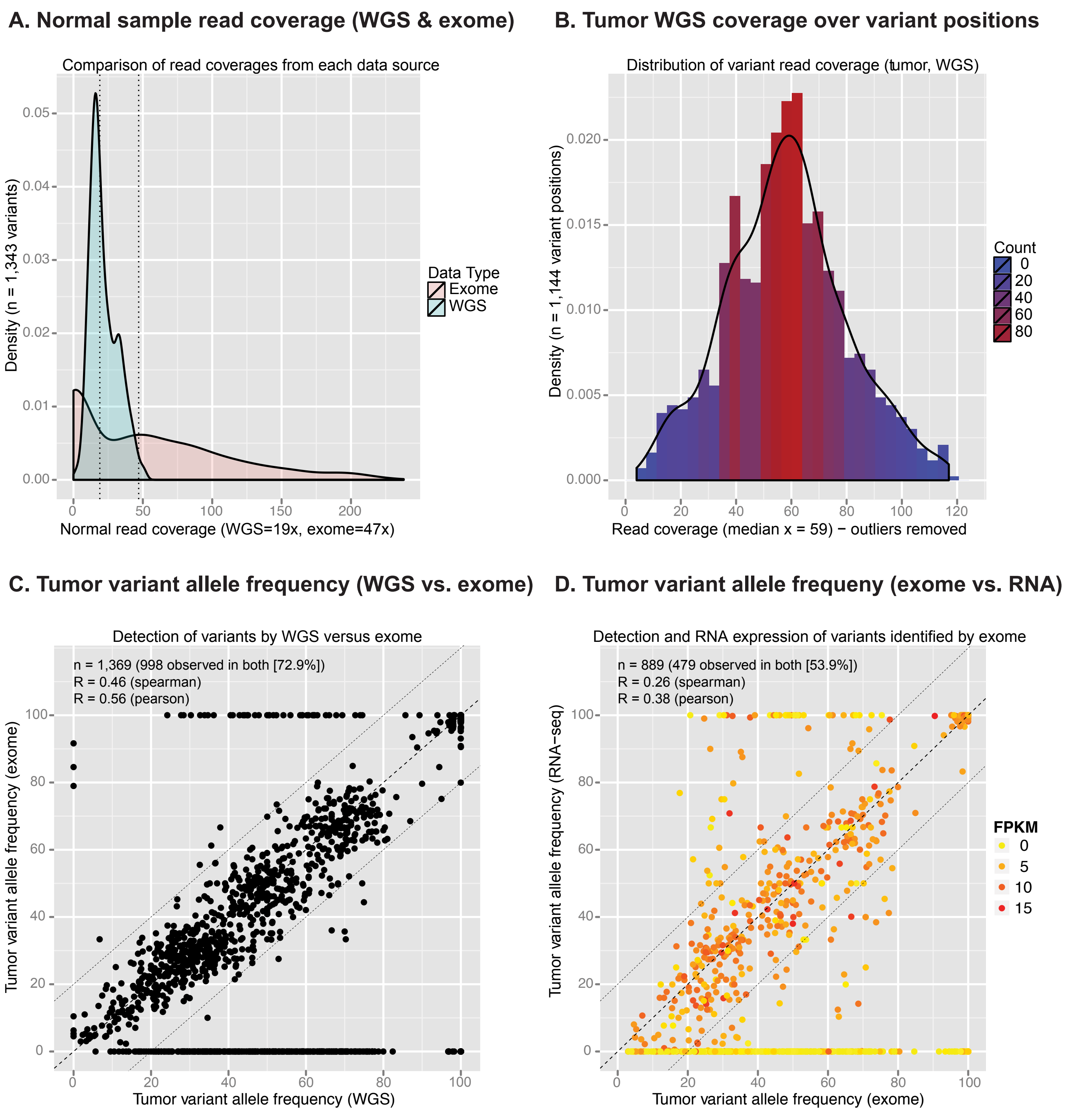

A. Normal sample read coverage (WGS & exome)
Comparison of read coverages from each data source
Density (n = 1,343 variants)
0.00
0.01
0.02
0.03
0.04
0.05
0
50
100
150
200
Normal read coverage (WGS=19x, exome=47x)
Data Type
Exome
WGS
B. Tumor WGS coverage over variant positions
Distribution of variant read coverage (tumor, WGS)
Density (n = 1,144 variant positions)
0.000
0.005
0.010
0.015
0.020
0
20
40
60
80
100
120
Read coverage (median x = 59) − outliers removed
Count
0
20
40
60
80
C. Tumor variant allele frequency (WGS vs. exome)
Detection of variants by WGS versus exome
n = 1,369 (998 observed in both [72.9%])
R = 0.46 (spearman)
R = 0.56 (pearson)
Tumor variant allele frequency (exome)
Tumor variant allele frequency (WGS)
0
20
40
60
80
100
D. Tumor variant allele frequeny (exome vs. RNA)
Detection and RNA expression of variants identified by exome
n = 889 (479 observed in both [53.9%])
R = 0.26 (spearman)
R = 0.38 (pearson)
Tumor variant allele frequency (RNA−seq)
Tumor variant allele frequency (exome)
0
20
40
60
80
100
FPKM
0
5
10
15
