# Supplementary material for: Genome Modeling System: A Knowledge Management Platform for Genomics
Source: PLoS Comput Biol. 2015 Jul 9;11(7):e1004274. doi: 10.1371/journal.pcbi.1004274 (PMC4497734; doi:10.1371/journal.pcbi.1004274)

A. Breakpoint encompassing read support for somatic HCC1395 gene fusions

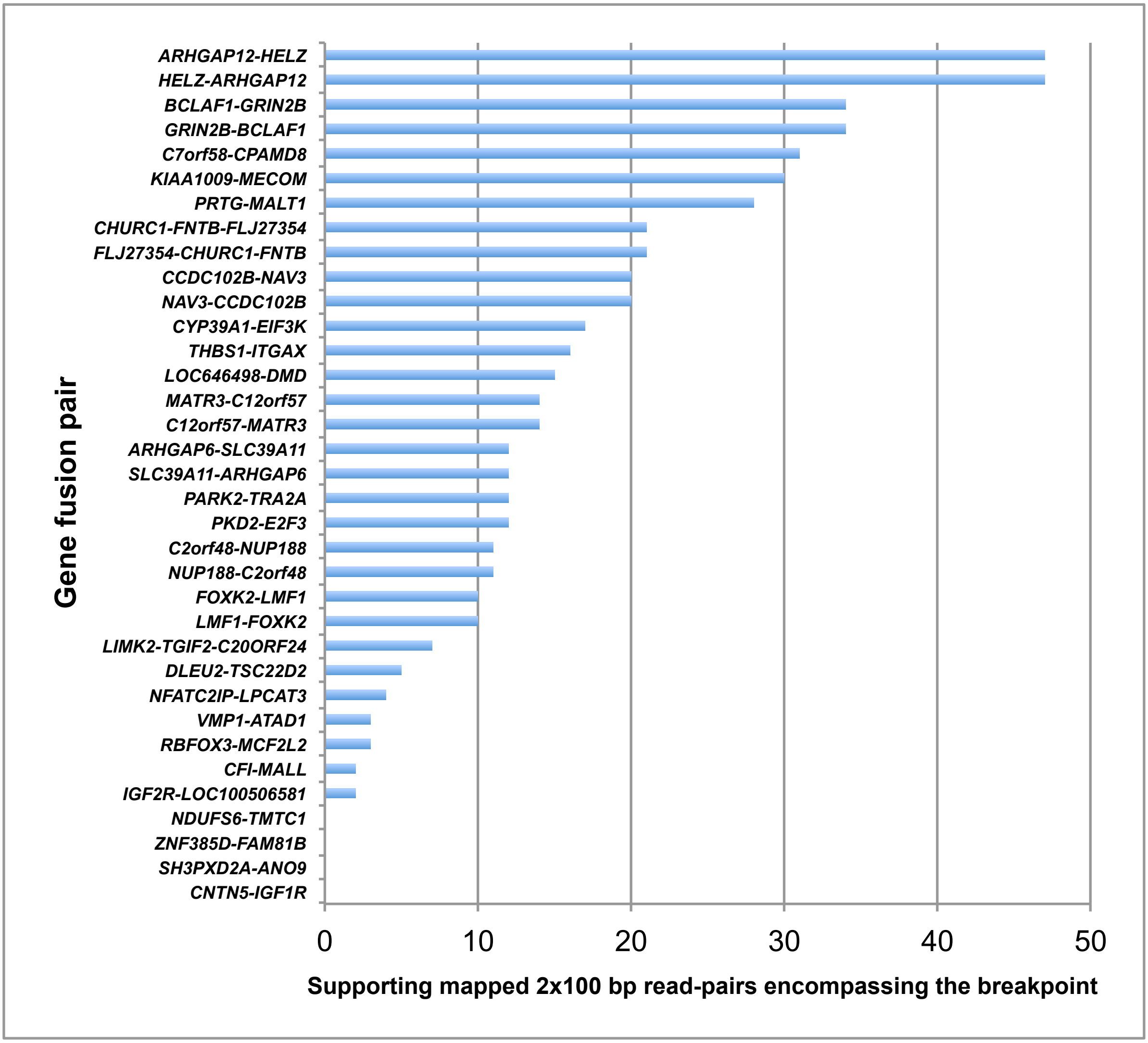

B. Coverage and breakpoint encompassing read support for: *PRTG-MALT1* fusion

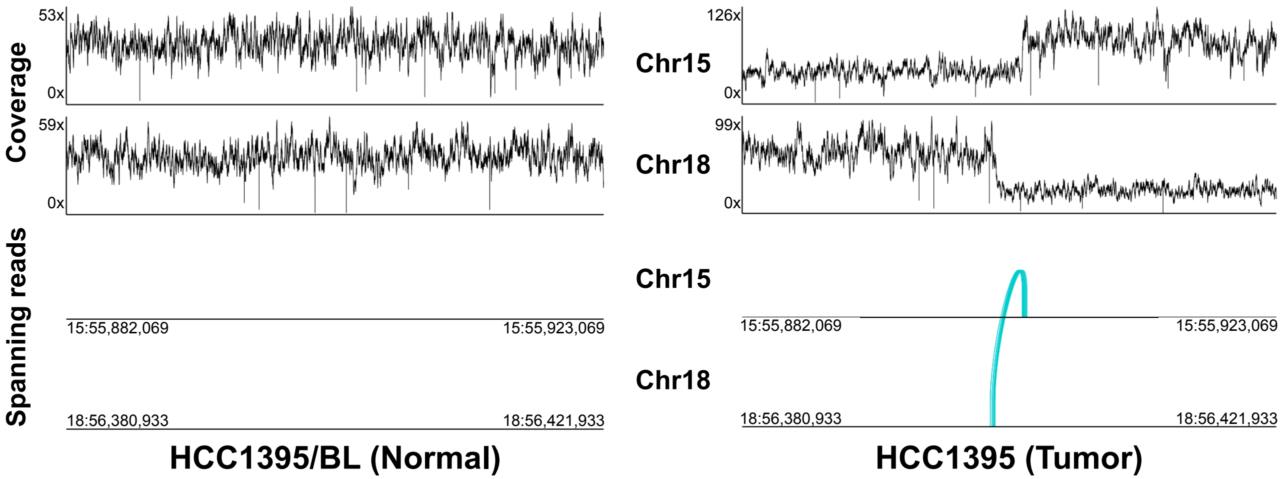

Supplement: S9 Fig — (A) A list of putative ORF maintaining gene fusions detected with the SV pipeline using BreakDancer [43] and SquareDancer are provided as a bar plot indicating the number of supporting discordant read pairs. (B) A ‘pairoscope’ plot illustrates the supporting reads for one of these potential fusions between PRTG and MALT1 on chromosomes 15 and 18. The complete list of predicted SVs from BreakDancer is provided as S4 Data. (PDF) [file pcbi.1004274.s009.pdf]
