# Supplementary material for: Genome Modeling System: A Knowledge Management Platform for Genomics
Source: PLoS Comput Biol. 2015 Jul 9;11(7):e1004274. doi: 10.1371/journal.pcbi.1004274 (PMC4497734; doi:10.1371/journal.pcbi.1004274)
Supplement: S10 Fig — (A) A clonality plot, displaying the distribution of VAFs plotted against WGS sequencing coverage is provided as a kernel density plot. (B) To obtain a clonality plot that excludes regions of copy number alteration, VAFs were limited to those from selected regions of a chromosome 21 with a copy-neutral state. These regions are indicated as dotted boxes on a plot of chromosome positions against tumor-normal copy number difference, where a value of 0 represents no difference in copy number between tumor and normal. (PDF) [file pcbi.1004274.s010.pdf]

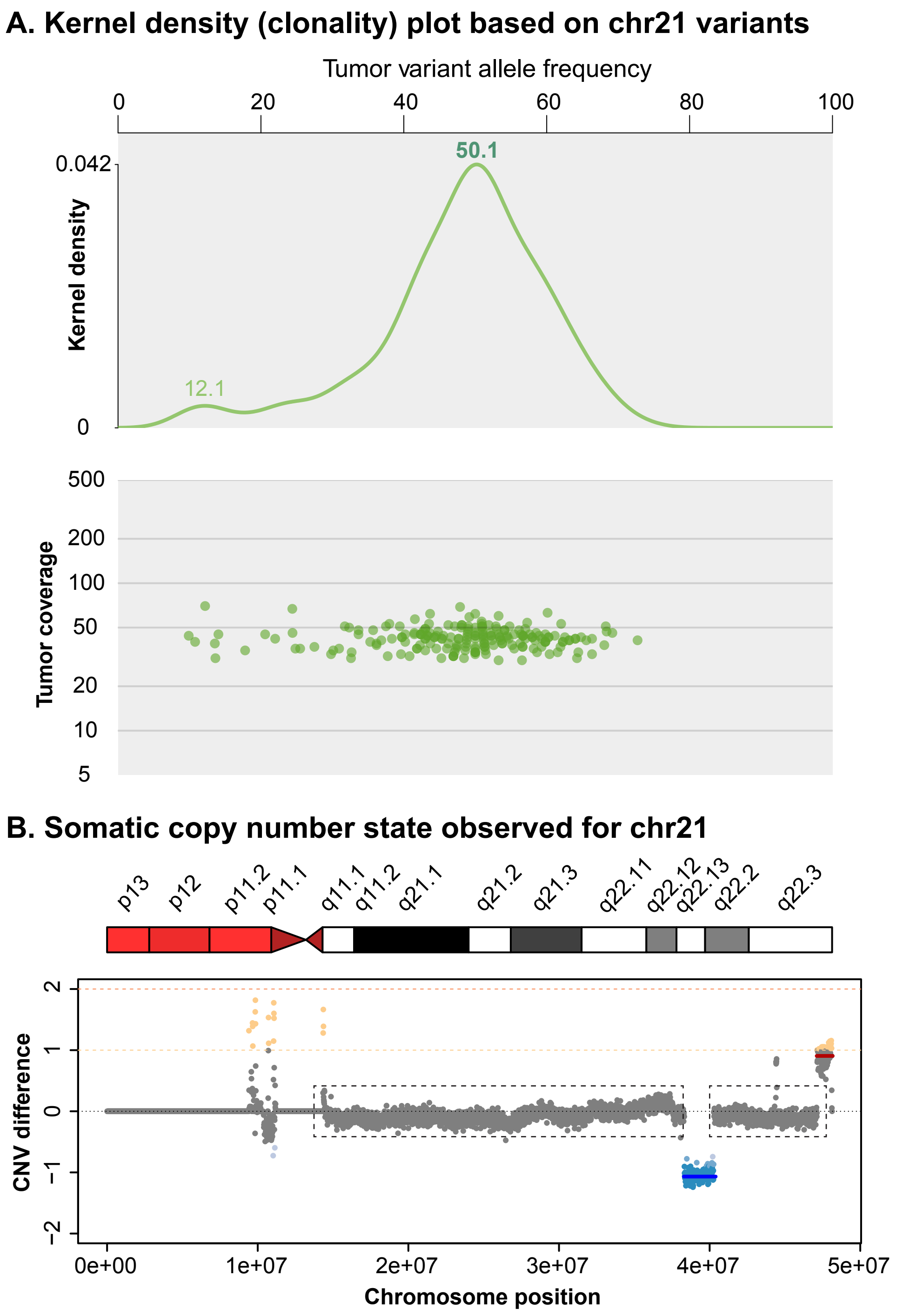
A. Kernel density (clonality) plot based on chr21 variants
Tumor variant allele frequency
0
20
40
60
80
100
Kernel density
0.042
0
50.1
12.1
Tumor coverage
500
200
100
50
20
10
5
B. Somatic copy number state observed for chr21
p13
p12
p11.2
p11.1
q11.1
q11.2
q21.1
q21.2
q21.3
q22.11
q22.12
q22.13
q22.2
q22.3
CNV difference
2
1
0
−1
−2
0e+00
1e+07
2e+07
3e+07
4e+07
5e+07
Chromosome position
