# Supplementary material for: Genome Modeling System: A Knowledge Management Platform for Genomics
Source: PLoS Comput Biol. 2015 Jul 9;11(7):e1004274. doi: 10.1371/journal.pcbi.1004274 (PMC4497734; doi:10.1371/journal.pcbi.1004274)
Supplement: S11 Fig — A sample of graphs automatically generated by the GMS to interpret RNA-seq results. (A) Library quality assessed by observed insert size distribution. (B) End bias plots showing the distribution of RNA-seq reads across the length of sequenced transcripts. (C) Percentage of reads aligning to the expected transcribed and non-transcribed regions. (D) Sequence coverage of known exon-exon junctions. (E) The observed patterns of splice site usage (F) The expression of an individual gene, NPM1, compared to the overall distribution of gene expression values. (PDF) [file pcbi.1004274.s011.pdf]

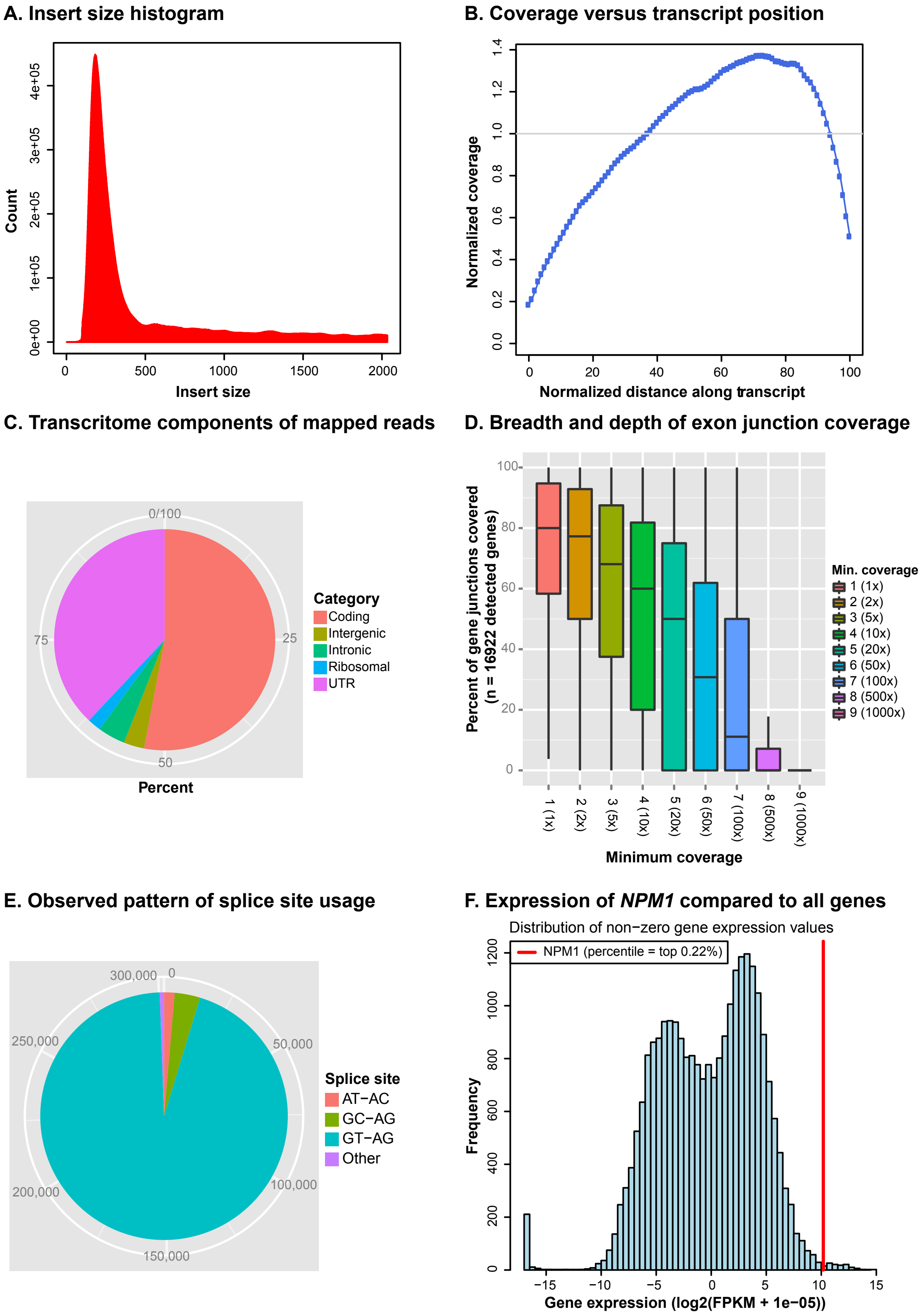

A. Insert size histogram
Count
Insert size
0e+00
1e+05
2e+05
3e+05
4e+05
0
500
1000
1500
2000
B. Coverage versus transcript position
Normalized coverage
Normalized distance along transcript
0.0
0.2
0.4
0.6
0.8
1.0
1.2
1.4
0
20
40
60
80
100
C. Transcritome components of mapped reads
0/100
25
50
75
Percent
Category
Coding
Intergenic
Intronic
Ribosomal
UTR
D. Breadth and depth of exon junction coverage
Percent of gene junctions covered (n = 16922 detected genes)
Minimum coverage
0
20
40
60
80
100
Min. coverage
1 (1x)
2 (2x)
3 (5x)
4 (10x)
5 (20x)
6 (50x)
7 (100x)
8 (500x)
9 (1000x)
E. Observed pattern of splice site usage
0
50,000
100,000
150,000
200,000
250,000
300,000
Splice site
AT−AC
GC−AG
GT−AG
Other
F. Expression of NPM1 compared to all genes
Distribution of non−zero gene expression values
NPM1 (percentile = top 0.22%)
Frequency
Gene expression (log2(FPKM + 1e−05))
0
200
400
600
800
1000
1200
−15
−10
−5
0
5
10
15
