# Supplementary material for: Genome Modeling System: A Knowledge Management Platform for Genomics
Source: PLoS Comput Biol. 2015 Jul 9;11(7):e1004274. doi: 10.1371/journal.pcbi.1004274 (PMC4497734; doi:10.1371/journal.pcbi.1004274)
Supplement: S13 Fig — The “genome model build view” command displays the status of all of the tasks within a build workflow. The following images show the build process for the WGS somatic variation build used in the example analysis. This is the same workflow illustrated in Fig 3. Image (A) shows the header for the build report, including the name of the model, the user who launched the build, and the ID for the processing profile. A table of steps is then presented. Each step has a database identifier, and also an ID for the job in the cluster management system (LSF). The status of the job is indicated in color. Where steps are nested, indentation of the name is used to suggest the situation visually. Variant detectors such as Pindel (A) and Breakdancer (B) have subordinate workflows, dividing work by genomic region. The TIGRA in silico SV validation step also divides work by chromosome (B, C and D), and is performed for each SV detection approach. For this build, the execution of VarScan2 and Strelka “shortcut” (B), indicating that the data set required already exists for the same inputs and parameters, presumably because of a prior build performing work with some overlap. The end of the report shows steps that merge results across approaches, and perform final annotation of variants. (PDF) [file pcbi.1004274.s013.pdf]

A. GMS build report header

ssmith — ssmith@blade14-2-16

```
ssmith@blade14-2-16 ~> genome model build view id=135798051
'build' may require verification...
Resolving parameter 'build' from command argument 'id=135798051'... found 1
=== Build ===
Build ID: 135798051                        Build Status: Succeeded
Model ID: 2891454547                       Model Name: H_NJ-HCC1395-HCC1395.somatic_variation-1
Run by: mgriffit                           Processing Profile ID: 2762562
Build Scheduled: 2013-03-18 14:52:49       Build Completed: 2013-03-20 23:38:04

Build Class: Genome::Model::Build::SomaticVariation
Software Revision: /gsc/scripts/opt/genome/snapshots/genome-3308/lib/perl:/etc/perl:/usr/local/lib/site_perl
SoftwareResult Test Name(s): undef (9)
Data Directory: /gscmnt/gc13001/info/model_data/2891454547/build135798051

=== Workflow ===
ID: 43829885                         Name: 135798051 Somatic Variation Pipeline
Started: 2013-03-18 14:52:59         Ended: 2013-03-20 23:37:58
User: mgriffit                       Cache ID: 2743347

ID        Status   LSF ID     Start Time           Time Elapsed   Name
43829885  done                2013-03-18 14:52:59  2d 08:44:59  135798051 Somatic Variation Pipeline
43829888  done     369510     2013-03-18 14:53:07  2d 07:36:00    Detect Variants
43829906  done                2013-03-18 14:53:16  2d 06:27:23    DetectVariants2 Dispatcher
43829909  done     396424     2013-03-18 20:16:40  1d 22:58:22      indel varscan-somatic 2.2.6 #1
43829915  done     396409     2013-03-18 20:16:35     06:54:01      indel strelka 0.4.6.2 #3
43829917  done     369743     2013-03-18 14:53:51     06:32:16      cnv plot-cnv v1 #1
43829946  done                2013-03-18 14:54:03     06:31:41      Plot Segments From Bams
43829950  done     369766     2013-03-18 14:54:11     04:22:54        normal bam-to-cn
43829949  done     369767     2013-03-18 14:54:12     06:04:15        tumor bam-to-cn
43829952  done     392434     2013-03-18 19:18:13     00:17:13        normal cnv-seg
43829951  done     399503     2013-03-18 20:59:35     00:24:00        tumor cnv-seg
43829953  done     399677     2013-03-18 21:24:42     00:00:02        plot segments
43829918  done     369702     2013-03-18 14:53:33     06:08:27      snv sniper 1.0.2 #4
43829921  done     shortcut   2013-03-18 14:53:54     05:22:25      indel pindel 0.5 #1
43829954  done                2013-03-18 14:54:08     03:35:22      Pindel Detect Variants Module
43829957  done     369772     2013-03-18 14:54:17     03:34:12        Pindel
43829958  done     370210     2013-03-18 14:56:54     03:04:12        Pindel
43829959  done     370216     2013-03-18 14:57:19     02:14:10        Pindel
43829960  done     370222     2013-03-18 14:56:59     02:26:31        Pindel
43829961  done     370224     2013-03-18 14:57:00     02:02:54        Pindel
43829962  done     370228     2013-03-18 14:57:06     01:54:38        Pindel
43829963  done     370236     2013-03-18 14:57:05     03:08:22        Pindel
43829964  done     370237     2013-03-18 14:57:10     01:48:18        Pindel
43829965  done     370160     2013-03-18 14:56:32     01:17:04        Pindel
43829966  done     370163     2013-03-18 14:56:31     02:04:55        Pindel
43829967  done     370174     2013-03-18 14:57:01     01:33:52        Pindel
43829968  done     370177     2013-03-18 14:56:37     01:26:38        Pindel
43829969  done     370181     2013-03-18 14:56:40     01:04:19        Pindel
43829970  done     370185     2013-03-18 14:56:42     01:13:07        Pindel
43829971  done     370188     2013-03-18 14:56:43     00:58:46        Pindel
43829972  done     370193     2013-03-18 14:56:44     01:02:21        Pindel
43829973  done     370198     2013-03-18 14:56:48     01:05:06        Pindel
43829974  done     370201     2013-03-18 14:56:49     01:11:05        Pindel
43829975  done     370206     2013-03-18 14:56:49     01:05:41        Pindel
43829976  done     370208     2013-03-18 14:56:55     00:49:58        Pindel
43829977  done     370114     2013-03-18 14:56:10     00:35:44        Pindel
43829978  done     370115     2013-03-18 14:56:09     00:32:06        Pindel
43829979  done     370119     2013-03-18 14:56:09     00:50:56        Pindel
43829980  done     370124     2013-03-18 14:56:13     00:04:43        Pindel
43829981  done     370129     2013-03-18 14:56:15     00:01:11        Pindel
```

B. GMS build report, subordinate workflows

```
ssmith — ssmith@blade14-2-16: ~ — ssh — 204×60
43830040  done      369827    2013-03-18 14:54:46     00:06:17      Pindel
43849761  done                2013-03-18 19:55:15     00:20:56    Multi-Vcf Merge
43849764  done      394763    2013-03-18 19:55:23     00:19:48      Pindel2Vcf
43829925  done      369718    2013-03-18 14:53:40     12:27:05    sv breakdancer 1.3 #7
43829928  done      369726    2013-03-18 14:53:40     01:03:17    sv breakdancer 1.3 #8
43830058  done      370086    2013-03-18 14:55:51     00:57:30    Breakdancer by chromosome
43830059  done      370162    2013-03-18 14:56:31     00:58:35      Breakdancer by chromosome
43830060  done      370175    2013-03-18 14:56:40     00:38:22      Breakdancer by chromosome
43830061  done      370179    2013-03-18 14:56:51     00:56:15      Breakdancer by chromosome
43830062  done      370186    2013-03-18 14:56:43     00:44:13      Breakdancer by chromosome
43830063  done      370192    2013-03-18 14:56:42     00:33:07      Breakdancer by chromosome
43830064  done      370199    2013-03-18 14:56:48     00:47:26      Breakdancer by chromosome
43830065  done      370202    2013-03-18 14:56:48     00:31:28      Breakdancer by chromosome
43830066  done      370207    2013-03-18 14:56:55     00:29:24      Breakdancer by chromosome
43830067  done      370211    2013-03-18 14:56:54     00:27:43      Breakdancer by chromosome
43830068  done      370219    2013-03-18 14:57:00     00:32:57      Breakdancer by chromosome
43830069  done      370226    2013-03-18 14:56:59     00:31:53      Breakdancer by chromosome
43830070  done      370095    2013-03-18 14:55:56     00:21:45      Breakdancer by chromosome
43830071  done      370098    2013-03-18 14:55:58     00:21:20      Breakdancer by chromosome
43830072  done      370105    2013-03-18 14:56:02     00:28:35      Breakdancer by chromosome
43830073  done      370111    2013-03-18 14:56:01     00:36:22      Breakdancer by chromosome
43830074  done      370116    2013-03-18 14:56:07     00:21:00      Breakdancer by chromosome
43830075  done      370120    2013-03-18 14:56:10     00:15:58      Breakdancer by chromosome
43830076  done      370125    2013-03-18 14:56:16     00:14:26      Breakdancer by chromosome
43830077  done      370130    2013-03-18 14:56:15     00:13:03      Breakdancer by chromosome
43830078  done      370135    2013-03-18 14:56:21     00:07:22      Breakdancer by chromosome
43830079  done      370137    2013-03-18 14:56:19     00:12:09      Breakdancer by chromosome
43830080  done      370148    2013-03-18 14:56:25     00:21:32      Breakdancer by chromosome
43830081  done      370150    2013-03-18 14:56:26     00:01:10      Breakdancer by chromosome
43830082  done      370158    2013-03-18 14:56:32     00:01:31      Breakdancer by chromosome
43829930  done      369528    2013-03-18 14:53:27     02:37:40    cnv bam-to-cna v1 #1
43829931  done      369688    2013-03-18 14:53:26     10:13:17    snv samtools r963 #9
43829933  done      369739    2013-03-18 14:53:49     12:01:33    sv squaredancer 0.1 #1
43829935  done      369746    2013-03-18 14:53:56     11:39:44    indel gatk-somatic-indel 5336 #1
43829910  done      496673    2013-03-20 19:16:12     00:02:24    indel_varscan-somatic_2.2.6_#1 varscan-high-confidence-indel v1 #1
43829912  done      shortcut  2013-03-20 19:16:10     00:00:01    snv varscan-somatic 2.2.6 #1
43829916  done      shortcut  2013-03-19 03:11:37     00:00:01    snv strelka 0.4.6.2 #3
43829919  done      399513    2013-03-18 21:03:07     15:48:15    snv_sniper_1.0.2_#4 false-positive v1 #2
43829922  done      396360    2013-03-18 20:16:29     02:03:13    indel_pindel_0.5_#1 pindel-somatic-calls v1 #1
43829926  done      404436    2013-03-19 03:21:54     05:27:52    sv_breakdancer_1.3_#7 novo-realign v1 #1
43829929  done      396384    2013-03-18 20:16:36     06:23:13    sv_breakdancer_1.3_#8 tigra-validation v1 #1
43852074  done      396561    2013-03-18 20:17:04     04:45:38    Tigra by chromosome
43852075  done      396902    2013-03-18 20:17:55     04:49:45      Tigra by chromosome
43852076  done      396908    2013-03-18 20:18:01     03:25:31      Tigra by chromosome
43852077  done      396925    2013-03-18 20:18:02     05:29:37      Tigra by chromosome
43852078  done      396938    2013-03-18 20:18:07     02:56:47      Tigra by chromosome
43852079  done      396943    2013-03-18 20:18:09     02:53:05      Tigra by chromosome
43852080  done      396969    2013-03-18 20:18:13     06:18:47      Tigra by chromosome
43852081  done      396989    2013-03-18 20:18:12     03:16:47      Tigra by chromosome
43852082  done      397013    2013-03-18 20:18:19     02:10:56      Tigra by chromosome
43852083  done      397040    2013-03-18 20:18:24     01:51:11      Tigra by chromosome
43852084  done      396578    2013-03-18 20:17:08     01:37:22      Tigra by chromosome
43852085  done      396598    2013-03-18 20:17:11     01:55:34      Tigra by chromosome
43852086  done      396603    2013-03-18 20:17:15     01:11:36      Tigra by chromosome
43852087  done      396633    2013-03-18 20:17:20     03:05:45      Tigra by chromosome
43852088  done      396656    2013-03-18 20:17:20     01:26:17      Tigra by chromosome
43852089  done      396671    2013-03-18 20:17:26     03:33:39      Tigra by chromosome
43852090  done      396703    2013-03-18 20:17:27     01:30:20      Tigra by chromosome
43852091  done      396725    2013-03-18 20:17:35     01:35:22      Tigra by chromosome
43852092  done      396745    2013-03-18 20:17:37     00:48:28      Tigra by chromosome
```

C.GMS build report, subordinate workflows - continued

ssmith — ssmith@blade14-2-16: ~ — ssh — 204×60

43852092	done	396745	2013-03-18 20:17:37	00:48:28	Tigra by chromosome
43852093	done	396764	2013-03-18 20:17:37	01:04:45	Tigra by chromosome
43852094	done	396782	2013-03-18 20:17:44	00:31:32	Tigra by chromosome
43852095	done	396789	2013-03-18 20:17:43	01:06:08	Tigra by chromosome
43852096	done	396805	2013-03-18 20:17:49	01:05:51	Tigra by chromosome
43852097	done	396835	2013-03-18 20:17:50	00:15:24	Tigra by chromosome
43852098	done	396874	2013-03-18 20:17:55	00:00:22	Tigra by chromosome
43829942	done	399684	2013-03-18 21:27:15	00:00:10	union cnv_bam-to-cna_v1_#1 cnv_plot-cnv_v1_#1
43829932	done	403802	2013-03-19 01:07:53	00:09:40	snv_samtools_r963_#9 snp-filter v1 #1
43829934	done	404327	2013-03-19 02:56:29	00:40:30	sv_squaredancer_0.1_#1 tigra-validation v1 #1
43858170	done	404328	2013-03-19 02:56:46	00:21:15	Tigra by chromosome
43858171	done	404345	2013-03-19 02:57:39	00:20:01	Tigra by chromosome
43858172	done	404346	2013-03-19 02:57:40	00:22:45	Tigra by chromosome
43858173	done	404347	2013-03-19 02:57:47	00:36:29	Tigra by chromosome
43858174	done	404348	2013-03-19 02:57:46	00:31:16	Tigra by chromosome
43858175	done	404349	2013-03-19 02:57:51	00:33:13	Tigra by chromosome
43858176	done	404350	2013-03-19 02:57:53	00:35:58	Tigra by chromosome
43858177	done	404351	2013-03-19 02:58:00	00:25:52	Tigra by chromosome
43858178	done	404353	2013-03-19 02:58:04	00:24:43	Tigra by chromosome
43858179	done	404354	2013-03-19 02:58:04	00:24:24	Tigra by chromosome
43858180	done	404329	2013-03-19 02:56:53	00:25:46	Tigra by chromosome
43858181	done	404330	2013-03-19 02:56:52	00:21:43	Tigra by chromosome
43858182	done	404331	2013-03-19 02:56:58	00:18:45	Tigra by chromosome
43858183	done	404332	2013-03-19 02:56:58	00:25:23	Tigra by chromosome
43858184	done	404333	2013-03-19 02:57:04	00:18:26	Tigra by chromosome
43858185	done	404334	2013-03-19 02:57:04	00:17:43	Tigra by chromosome
43858186	done	404335	2013-03-19 02:57:10	00:24:55	Tigra by chromosome
43858187	done	404336	2013-03-19 02:57:10	00:20:43	Tigra by chromosome
43858188	done	404337	2013-03-19 02:57:18	00:24:49	Tigra by chromosome
43858189	done	404338	2013-03-19 02:57:21	00:16:24	Tigra by chromosome
43858190	done	404339	2013-03-19 02:57:21	00:11:47	Tigra by chromosome
43858191	done	404340	2013-03-19 02:57:27	00:11:23	Tigra by chromosome
43858192	done	404341	2013-03-19 02:57:28	00:12:26	Tigra by chromosome
43858193	done	404342	2013-03-19 02:57:34	00:06:31	Tigra by chromosome
43858194	done	404344	2013-03-19 02:57:34	00:00:55	Tigra by chromosome
43829936	done	404035	2013-03-19 02:34:51	00:30:24	indel_gatk-somatic-indel_5336_#1 false-indel v1 #2
43829911	done	496870	2013-03-20 19:19:46	00:59:39	indel_varscan-somatic_2.2.6_#1 false-indel v1 #2
43829913	done	496678	2013-03-20 19:16:23	00:09:22	snv_varscan-somatic_2.2.6_#1 varscan-high-confidence v1 #1
43829920	done	416781	2013-03-19 12:52:31	03:43:40	snv_sniper_1.0.2_#4 somatic-score-mapping-quality v1 #5
43829923	done	401439	2013-03-18 22:20:54	06:19:16	indel_pindel_0.5_#1 pindel-vaf-filter v1 #6
43829927	done	407519	2013-03-19 08:50:56	01:27:34	sv_breakdancer_1.3_#7 tigra-validation v1 #1
43859105	done	407521	2013-03-19 08:51:18	00:05:46	Tigra by chromosome
43859106	done	407533	2013-03-19 08:51:53	00:08:34	Tigra by chromosome
43859107	done	407534	2013-03-19 08:51:58	00:08:57	Tigra by chromosome
43859108	done	407552	2013-03-19 08:52:03	00:10:08	Tigra by chromosome
43859109	done	407571	2013-03-19 08:52:04	00:19:26	Tigra by chromosome
43859110	done	407572	2013-03-19 08:52:09	00:26:57	Tigra by chromosome
43859111	done	407573	2013-03-19 08:52:09	00:23:08	Tigra by chromosome
43859112	done	407574	2013-03-19 08:52:15	00:14:33	Tigra by chromosome
43859113	done	407575	2013-03-19 08:52:15	00:43:36	Tigra by chromosome
43859114	done	407576	2013-03-19 08:52:21	00:25:30	Tigra by chromosome
43859115	done	407577	2013-03-19 08:52:21	00:45:56	Tigra by chromosome
43859116	done	407578	2013-03-19 08:52:26	00:11:06	Tigra by chromosome
43859117	done	407579	2013-03-19 08:52:32	00:20:08	Tigra by chromosome
43859118	done	407581	2013-03-19 08:52:32	00:19:56	Tigra by chromosome
43859119	done	407522	2013-03-19 08:51:18	00:56:51	Tigra by chromosome
43859120	done	407523	2013-03-19 08:51:24	00:28:05	Tigra by chromosome
43859121	done	407524	2013-03-19 08:51:30	00:29:05	Tigra by chromosome
43859122	done	407526	2013-03-19 08:51:29	00:21:40	Tigra by chromosome
43859123	done	407527	2013-03-19 08:51:36	00:42:34	Tigra by chromosome

D. GMS build report, subordinate workflows - continued

```
ssmith — ssmith@blade14-2-16: ~ — ssh — 204×60
43858181  done    404330    2013-03-19 02:56:52    00:21:43      Tigra by chromosome
43858182  done    404331    2013-03-19 02:56:58    00:18:45      Tigra by chromosome
43858183  done    404332    2013-03-19 02:56:58    00:25:23      Tigra by chromosome
43858184  done    404333    2013-03-19 02:57:04    00:18:26      Tigra by chromosome
43858185  done    404334    2013-03-19 02:57:04    00:17:43      Tigra by chromosome
43858186  done    404335    2013-03-19 02:57:10    00:24:55      Tigra by chromosome
43858187  done    404336    2013-03-19 02:57:10    00:20:43      Tigra by chromosome
43858188  done    404337    2013-03-19 02:57:18    00:24:49      Tigra by chromosome
43858189  done    404338    2013-03-19 02:57:21    00:16:24      Tigra by chromosome
43858190  done    404339    2013-03-19 02:57:21    00:11:47      Tigra by chromosome
43858191  done    404340    2013-03-19 02:57:27    00:11:23      Tigra by chromosome
43858192  done    404341    2013-03-19 02:57:28    00:12:26      Tigra by chromosome
43858193  done    404342    2013-03-19 02:57:34    00:06:31      Tigra by chromosome
43858194  done    404344    2013-03-19 02:57:34    00:00:55      Tigra by chromosome
43829936  done    404035    2013-03-19 02:34:51    00:30:24    indel_gatk-somatic-indel_5336_#1 false-indel v1 #2
43829911  done    496870    2013-03-20 19:19:46    00:59:39    indel_varscan-somatic_2.2.6_#1 false-indel v1 #2
43829913  done    496678    2013-03-20 19:16:23    00:09:22    snv_varscan-somatic_2.2.6_#1 varscan-high-confidence v1 #1
43829920  done    416781    2013-03-19 12:52:31    03:43:40    snv_sniper_1.0.2_#4 somatic-score-mapping-quality v1 #5
43829923  done    401439    2013-03-18 22:20:54    06:19:16    indel_pindel_0.5_#1 pindel-vaf-filter v1 #6
43829927  done    407519    2013-03-19 08:50:56    01:27:34    sv_breakdancer_1.3_#7 tigra-validation v1 #1
43859105  done    407521    2013-03-19 08:51:18    00:05:46    Tigra by chromosome
43859106  done    407533    2013-03-19 08:51:53    00:08:34      Tigra by chromosome
43859107  done    407534    2013-03-19 08:51:58    00:08:57      Tigra by chromosome
43859108  done    407552    2013-03-19 08:52:03    00:10:08      Tigra by chromosome
43859109  done    407571    2013-03-19 08:52:04    00:19:26      Tigra by chromosome
43859110  done    407572    2013-03-19 08:52:09    00:26:57      Tigra by chromosome
43859111  done    407573    2013-03-19 08:52:09    00:23:08      Tigra by chromosome
43859112  done    407574    2013-03-19 08:52:15    00:14:33      Tigra by chromosome
43859113  done    407575    2013-03-19 08:52:15    00:43:36      Tigra by chromosome
43859114  done    407576    2013-03-19 08:52:21    00:25:30      Tigra by chromosome
43859115  done    407577    2013-03-19 08:52:21    00:45:56      Tigra by chromosome
43859116  done    407578    2013-03-19 08:52:26    00:11:06      Tigra by chromosome
43859117  done    407579    2013-03-19 08:52:32    00:20:08      Tigra by chromosome
43859118  done    407581    2013-03-19 08:52:32    00:19:56      Tigra by chromosome
43859119  done    407522    2013-03-19 08:51:18    00:56:51      Tigra by chromosome
43859120  done    407523    2013-03-19 08:51:24    00:28:05      Tigra by chromosome
43859121  done    407524    2013-03-19 08:51:30    00:29:05      Tigra by chromosome
43859122  done    407526    2013-03-19 08:51:29    00:21:40      Tigra by chromosome
43859123  done    407527    2013-03-19 08:51:36    00:42:34      Tigra by chromosome
43859124  done    407528    2013-03-19 08:51:38    01:24:19      Tigra by chromosome
43859125  done    407529    2013-03-19 08:51:41    00:22:32      Tigra by chromosome
43859126  done    407530    2013-03-19 08:51:41    00:28:46      Tigra by chromosome
43859127  done    407531    2013-03-19 08:51:47    00:35:58      Tigra by chromosome
43859128  done    407532    2013-03-19 08:51:52    00:00:28      Tigra by chromosome
43829939  done    501204    2013-03-20 20:20:34    00:01:00    unionunique indel_varscan-somatic_2.2.6_#1 indel_strelka_0.4.6.2_#3
43829914  done    497428    2013-03-20 19:26:54    01:29:08    snv_varscan-somatic_2.2.6_#1 false-positive v1 #2
43829943  done    432306    2013-03-19 16:37:16    00:06:07    intersect snv_samtools_r963_#9 snv_sniper_1.0.2_#4
43829924  done    405162    2013-03-19 04:41:23    00:38:45    indel_pindel_0.5_#1 pindel-read-support v1 #1
43829937  done    409395    2013-03-19 10:19:39    00:00:12    union sv_breakdancer_1.3_#7 sv_breakdancer_1.3_#8
43829944  done    502579    2013-03-20 20:57:15    00:06:20    unionunique snv_varscan-somatic_2.2.6_#1 snv_strelka_0.4.6.2_#3
43829940  done    501342    2013-03-20 20:22:43    00:03:44    unionunique indel_pindel_0.5_#1 unionunique-indel_varscan-somatic_2.2.6__1-indel_strelka_0.4.6.2__3
43829938  done    409439    2013-03-19 10:20:58    00:00:12    union union-sv_breakdancer_1.3__7-sv_breakdancer_1.3__8 sv_squaredancer_0.1_#1
43829945  done    503025    2013-03-20 21:04:45    00:14:54    unionunique intersect-snv_samtools_r963__9-snv_sniper_1.0.2__4 unionunique-snv_varscan-somatic_2.2.6__1-snv_strelka_0.4.6.2__3
43829941  done    501611    2013-03-20 20:27:36    00:01:07    unionunique indel_gatk-somatic-indel_5336_#1 unionunique-indel_pindel_0.5__1-unionunique-indel_varscan-somatic_2.2.6__1-indel_strelka_
0.4.6.2__3
43829889  done    504072    2013-03-20 22:30:19    00:00:37   Identify LOH
43829890  done    504073    2013-03-20 22:32:11    00:00:11   Identify Previously Discovered Variations
43829891  done    504074    2013-03-20 22:33:32    00:02:04   Tier Variants
43829892  done    504078    2013-03-20 22:36:43    01:00:15   Annotate And Upload Variants
ssmith@blade14-2-16 ~>
```
