# Supplementary material for: Genome Modeling System: A Knowledge Management Platform for Genomics
Source: PLoS Comput Biol. 2015 Jul 9;11(7):e1004274. doi: 10.1371/journal.pcbi.1004274 (PMC4497734; doi:10.1371/journal.pcbi.1004274)
Supplement: S14 Fig — The GMS web search interface provides high-speed access to large volumes of data. (A) It offers separate tabs to allow searching by model, build, processing profiles, instrument data, or subject. The free-form search box provides direct access to querying the database without the analyst knowing exact field names and nomenclature. The results in each tab have links to the other related entities in the system, as well as the ability to drill down for additional detail about the entity in question. This example shows a search for models related to the HCC1395 cell line subject. (B) This page for an individual sample shows general data about the sample, followed by a link to information about DNA fragment libraries, behind which are specifics about instrument data. Below this page begins a list of models that have been made with this sample as the subject. (C) Each listed model shows its processing profile and inputs, as well as a list of build attempts, and respective build statuses. In this example, exome-capture based alignment and variant detection are running. The genotype microarray analysis of the same sample has completed successfully, but prior to that had one failed attempt at processing. (D) The fourth image shows details for a specific build, including a list of specific steps, and the status of each on the compute cluster. Links are present to the log files of each step, and also to the log file for the build process as a whole. (PDF) [file pcbi.1004274.s014.pdf]

A. Summary of existing builds

Genome Modeling System | GMS ID: RH9YB64

Models | Processing Profiles | Instrument Data | Subjects | Builds

10

records per page

Filter results:

Build	Status	Model
106942997	Succeeded	GRCh37-lite
108563338	Succeeded	nimblegen-human
124434505	Succeeded	NCBI-human.ensembl
127786607	Succeeded	dbsnp-GRCh37-lite-build37
27f279a6f9ff4e5990f9e5c57c7c009b	Succeeded	hcc1395-tumor-snparray
2a9668308dbc45ebb3629685f419b05c	Succeeded	hcc1395-normal-snparray
2fe02ce7e95d4af5932f822d441fdd77	Scheduled	hcc1395-tumor-snparray
4051270386394d26947c394225fbf68d	Succeeded	hcc1395-normal-snparray
45938db266f6481a9d3a17762b4da570	Scheduled	hcc1395-tumor-snparray
46e9f1eba1c84d69bc7c3947fce6dab2	Scheduled	hcc1395-normal-snparray

Showing 1 to 10 of 16 entries

← Previous | 1 | 2 | Next →

B. Sample view for a single sample

Genome Modeling System GMS ID: U6GNV74

H_NJ-HCC1395 sample

Attribute	Value
upn	HCC1395
ethnicity	Caucasian
nomenclature	GC
gender	female
taxon_id	1653198737
common_name	TST1

Taxon

human

C. Detailed summary for a single build

Genome Modeling System GMS ID: U6GNV74

173248c1921443eeb345f32c9930dd39

hcc1395-normal-refalign-exome build

Attribute	Value
status	Succeeded
user	ubuntu
model	2891377978
subject	2889981254
files	data errors output
processing_profile	Default Reference Alignment
workflow	173248c1921443eeb345f32c9930dd39 all stages (c6ad983597a442b9a478d61779b549f1)

Inputs

Attribute	Value
dbsnp_build	build 127786607
instrument_data	instrument data 2891351068
reference_sequence_build	build 106942997
genotype_microarray	build b17b849be38548fcb9196da3dcd61a30
region_of_interest_set_name	NimbleGen v3 Capture Chip Set
target_region_set_name	NimbleGen v3 Capture Chip Set
annotation_reference_build	build 124434505

D. Status of workflow steps

Genome Modeling System GMS ID: U6GNV74

Workflow

173248c1921443eeb345f32c9930dd39 all stages /

Name	Status	LSF id	Started At	Finished At	Elapsed	Stdout	Stderr
prepare-reference-sequence-index 1dfea28c59014b99923f86d9e23526bb	Done	P18468	2015-03-19 14:08:35 UTC	2015-03-19 14:08:37 UTC	less than a minute	Output	Errors
align-reads 047c4fc70a4840dea9f2e77f468a77bf	Done	114	2015-03-19 14:08:49 UTC	2015-03-19 14:11:19 UTC	3 minutes	Output	Errors
bam-qc b78b63ab98494207957e22384b3a4a12	Done	115	2015-03-19 14:12:30 UTC	2015-03-19 14:14:59 UTC	2 minutes	Output	Errors
merge-alignments 9a3eac7f71414af89baa470b7047c320	Done	121	2015-03-19 14:16:09 UTC	2015-03-19 14:16:35 UTC	less than a minute	Output	Errors
coverage-stats 0eea7a1a320f4f1bac091c4ede89d761	Done	122	2015-03-19 14:17:49 UTC	2015-03-19 14:48:51 UTC	31 minutes	Output	Errors
detect-variants 99f177ba53004341bfad14373084d540	Done	131	2015-03-19 14:49:59 UTC	2015-03-19 14:56:41 UTC	7 minutes	Output	Errors
annotate-adaptor 3c0fda69ec83491393f05a8dceb1d8a3	Done	135	2015-03-19 14:57:49 UTC	2015-03-19 14:57:52 UTC	less than a minute	Output	Errors
annotate-transcript-variants da69f86c212c4892b7d23ee3f30e507c	Done	136	2015-03-19 14:58:59 UTC	2015-03-19 15:02:07 UTC	3 minutes	Output	Errors
run-reports 1a7b459f5d0a4bda8ae7681ad6af43e5	Done	137	2015-03-19 15:03:20 UTC	2015-03-19 15:06:32 UTC	3 minutes	Output	Errors
