# Supplementary material for: Genome Modeling System: A Knowledge Management Platform for Genomics
Source: PLoS Comput Biol. 2015 Jul 9;11(7):e1004274. doi: 10.1371/journal.pcbi.1004274 (PMC4497734; doi:10.1371/journal.pcbi.1004274)
Supplement: S1 Table — (PDF) [file pcbi.1004274.s016.pdf]

S1 Table. HCC1395/BL whole genome (DNA) sequence metrics

Metric	Tumor	Normal
Library median insert sizes<br>(3 libraries were created for each DNA sample)	259 bp, 366 bp, 513 bp	252 bp, 358 bp, 522 bp
Read 1 / Read 2 average error rates	0.34 / 0.37	0.31 / 0.36
Total read count<br>(2 x 100 bp reads)	941,755,727	515,536,028
Sequence amount (gbp)	188.4	103.1
Haploid coverage	56x	31x
Mapped read percentage	95.2%	95.0%
Duplicate read percentage	5.3%	3.0%
WGS SNP versus microarray SNP concordance	98.7%	99.6%
WGS SNP versus dbSNP concordance	94.5%	96.2%

Average error rates are reported per base position in the read. Reads were mapped by BWA and duplicate read percentages were determined by Picard MarkDuplicates. Microarray SNP genotype concordance was determined by comparing genotypes from Illumina Infinium arrays to genotypes determined from the WGS sequence data. WGS 'SNP versus dbSNP' concordance refers to the percentage of variant positions observed in the sequence data that correspond to a known SNP from dbSNP.
