# Supplementary material for: Genome Modeling System: A Knowledge Management Platform for Genomics
Source: PLoS Comput Biol. 2015 Jul 9;11(7):e1004274. doi: 10.1371/journal.pcbi.1004274 (PMC4497734; doi:10.1371/journal.pcbi.1004274)
Supplement: S2 Table — (PDF) [file pcbi.1004274.s017.pdf]

S2 Table. HCC1395/BL whole exome (DNA) sequence metrics

Metric	Tumor	Normal
Library median insert size	239 bp	243 bp
Read 1/Read 2 average error rates	0.21 / 0.24	0.21 / 0.23
Total read count (2 x 100 bp reads)	96,056,889	77,667,085
Sequence amount (gbp)	19.2	15.5
Mapped read percentage	99.0%	99.0%
Percent exons sequenced >20X over >80% of base positions	95.6%	95.3%
Median coverage of targeted bases	155x	124x
