# Supplementary material for: Genome Modeling System: A Knowledge Management Platform for Genomics
Source: PLoS Comput Biol. 2015 Jul 9;11(7):e1004274. doi: 10.1371/journal.pcbi.1004274 (PMC4497734; doi:10.1371/journal.pcbi.1004274)
Supplement: S3 Table — (PDF) [file pcbi.1004274.s018.pdf]

S3 Table. HCC1395/BL transcriptome (RNA) sequence metrics

Metric	Tumor	Normal
Library median insert size	220 bp	281 bp
Total read count (2 x 100 bp reads)	156,248,832	170,049,877
Mapped read percentage	81.7%	78.1%
Percent coding bases	53.5%	56.4%
Percent UTR bases	38.3%	30.7%
Percent intronic bases	3.9%	5.6%
Percent intergenic bases	2.7%	3.0%
Percent ribosomal bases	1.7%	4.2%
Number of genes detected (FPKM >1)	12,846	13,281
Percentage of all Ensembl exon-exon junctions detected	54.4%	55.8%
Percentage of detected exon-exon junctions that are known to Ensembl	90.1%	91.5%
Percentage of reads consumed by the top 1% of expressed genes	49.4%	34.7%
