# Supplementary material for: Genome Modeling System: A Knowledge Management Platform for Genomics
Source: PLoS Comput Biol. 2015 Jul 9;11(7):e1004274. doi: 10.1371/journal.pcbi.1004274 (PMC4497734; doi:10.1371/journal.pcbi.1004274)
Supplement: S4 Table — (PDF) [file pcbi.1004274.s019.pdf]

S4 Table. HCC1395/BL selected candidate cancer associated SNVs

Gene	Amino Acid Change	WGS Tumor VAF	Exome Tumor VAF	RNA-seq Tumor VAF	RNA-seq Tumor gene FPKM	Mutant Expression Class
TP53	R175H	100.00	98.86	99.94	153.62	Expressed
PDGFRB	T882I	28.33	29.13	100.00	0.06	Silent Gene
FGFR1	S158L	100.00	100.00	100.00	51.12	Expressed
CREBBP	H1297Q	29.69	23.53	31.64	20.98	Expressed
BRCA2	E1593*	45.26	42.59	48.39	2.02	Expressed
ABL2	T753A	31.58	35.48	33.33	5.62	Expressed
MLL	C151F	9.30	7.46	0.00	5.71	Unknown
MSH6	D1255N	32.47	31.82	30.53	19.47	Expressed
EPHA8	N123K	36.36	26.72	0.00	0.00	Silent Gene
EPHA10	L709M	33.33	38.22	0.00	0.01	Silent Gene
EPHB1	E245*	9.38	5.94	0.00	0.04	Silent Gene
PLCG1	S582C	40.82	42.86	53.49	9.69	Expressed
CASC5	P865S	49.15	46.15	50.00	3.10	Expressed
STAT1	P493A	48.81	58.10	53.71	46.85	Expressed
TRPM7	K153T	47.37	50.00	44.95	3.45	Expressed
CFTR	G437C	13.30	10.94	0.00	0.00	Silent Gene
PRKX	V43A	100.00	100.00	100.00	18.92	Expressed
FLNA	D2581H	100.00	99.17	99.81	294.41	Expressed
SGK3	A92V	42.62	22.86	27.27	0.44	Expressed
