# Supplementary material for: Genome Modeling System: A Knowledge Management Platform for Genomics
Source: PLoS Comput Biol. 2015 Jul 9;11(7):e1004274. doi: 10.1371/journal.pcbi.1004274 (PMC4497734; doi:10.1371/journal.pcbi.1004274)
Supplement: S5 Table — (PDF) [file pcbi.1004274.s020.pdf]

S5 Table. Test hardware configurations

System	Hardware Specification	Cost	Operating System	Comments
MacBook Pro	4 CPUs, 16 Gb RAM, 750 Gb solid state storage	$3,000	OS X 10.9.2 with Ubuntu 12.04 installed via Vagrant + virtualbox)	Used to test installation
Dell Precision T7600	8 CPUs, 64 Gb RAM, 5 Tb SATA storage	$4,000	Ubuntu 12.04 Precise	Used to test installation, for network isolation testing and to run analysis with down-sampled data
MacPro Desktop	8 CPUs, 64Gb RAM, 7 Tb SATA storage	$6,000	OS X 10.9.2 with Ubuntu 12.04 installed via Vagrant + virtualbox)	Used to test multiple concurrent installations and run analysis with down-sampled data
Dell data center blade	24 CPUs, 128 Gb RAM, 2Tb+ (SATA + network disk)	$20,000	Ubuntu 12.04 Precise	Used for analysis of full HCC1395 WGS, exome and RNA-seq data
Dell Power Edge R910 4U	80 CPUs, 1 Tb RAM, 2.4 Tb Fusion IO storage	$53,000	Ubuntu 12.04 Precise	Used for analysis of full HCC1395 WGS, exome and RNA-seq data. Multiple samples and pipelines run concurrently
Amazon AWS EC2 Instances	i2.2xlarge, c3.8xlarge, r3.8xlarge, i2.4xlarge, i2.8xlarge, and hs1.8xlarge.	$1.68 to $6.82 per hour	Ubuntu 12.04 Precise (search for AMI: 'SGMS_INSTALL')	Used for various tests of HCC1395 data and other public data. Instance type can be tailored to the amount of data

All costs are approximate. For complete details of Amazon AWS EC2 instance type hardware specifications and costs please refer to: http://aws.amazon.com/ec2/pricing/
