# Supplementary material for: Genome Modeling System: A Knowledge Management Platform for Genomics
Source: PLoS Comput Biol. 2015 Jul 9;11(7):e1004274. doi: 10.1371/journal.pcbi.1004274 (PMC4497734; doi:10.1371/journal.pcbi.1004274)
Supplement: S6 Table — (PDF) [file pcbi.1004274.s021.pdf]

**S6 Table. The GMS, conceptually related resources, and their features**

| Name | Published | Source Code | Commercial | Open Source | Analysis Tracking | Pipelines |
|---|---|---|---|---|---|---|
| Genome Modeling System | Pending | Yes | No | Yes | Yes | G, S, R, D, C |
| Galaxy | Yes | Yes | No | Yes | Yes | G, S, R, D |
| bcbio-nextgen | No | Yes | No | Yes | No | G, S, R, D, C |
| Illumina BaseSpace | No | No | Yes | No | Yes | G, S, R, D, C |
| SeqWare | Yes | Yes | No | Yes | Yes | None |
| DNA Nexus Platform | No | Yes | Yes | Partial | Yes | G, S, R, C |
| gkno | No | Yes | No | Yes | No | G |
| GATK's Queue | No | Yes | Both | Partial | No | G, S, C |
| Appistry's Ayrris | No | No | Yes | No | No | G, S, C |
| Curoverse's Arvados | No | Yes | Both | Yes | Yes | G |
| CGA's Firehose | No | No | No | No | Yes | S, R, |
| Seven Bridges Genomics | No | No | Yes | No | Yes | G, R, D |
| ga4gh | No | Yes | Both | Yes | N/A | None |
| IBM's PowerGene Orchestrator | No | No | Yes | No | N/A | None |
| MIT STAR | Yes | Yes | No | Yes | No | None |

Abbreviations: G = Germline, S = Somatic variation, R = RNA-seq expression, D = Differential expression, C = Clinical interpretation

For a more detailed and up to date version of this table maintained online, please refer to: https://github.com/genome/gms/wiki/The-GMS-compared-to-conceptually-related-resources
