# Supplementary material for: Genome Modeling System: A Knowledge Management Platform for Genomics
Source: PLoS Comput Biol. 2015 Jul 9;11(7):e1004274. doi: 10.1371/journal.pcbi.1004274 (PMC4497734; doi:10.1371/journal.pcbi.1004274)
Supplement: S1 Text — (PDF) [file pcbi.1004274.s029.pdf]

Supplementary Methods

Tool tree and API

Components in the tool tree can be written in Perl, or in other languages such as R, Python, Ruby or Java with a small file describing essential meta-data. The meta-data for each component is a simple hash, and provides several features at once: automatic command-line interface generation, automatic tab-completion, dynamically generated documentation, input/output information for use in the workflow/build system, and a resource requirements specification for use in job scheduling on a compute cluster. The documentation generation for GMS components includes command-line help, manual pages, and web-based documentation for components when published on the Genome Modeling Tools web site (http://gmt.genome.wustl.edu). Automatically generated documentation is less prone to become outdated, since a component's documented parameters come from the same structure that specifies functionality to the command-line, and to the compiler/interpreter. Because the documents are composed of free-form sections that the author can add to each module, rich documentation is possible for complex tools.

Subjects and instrument data tracking

The GMS tracks input data used for analysis with two flexible metaphors "Subjects" and "Instrument Data" (**Box 1**). One unit of instrument data might be a lane of sequence data from an Illumina flow cell, a region of a 454 run, or the microarray results for a given sample. All instrument data are linked to a subject record for the DNA/RNA sample in question. The subject sample is linked to a subject individual from which the sample came. This, in turn, is linked to a subject taxonomic umbrella. Additional subjects can be defined for cohorts, with arbitrary control over addition and removal of members. With this simple system the GMS summarizes a subset of typical laboratory information management system (LIMS) information, exposing it to both manual and automated analysis. A command-line interface is available for manipulation of these data, and all data are discoverable in the web-based search engine as well. **Box 2** demonstrates the use of the bioinformatics command-line to manipulate subjects and instrument data, and to launch analysis.

Reference sequences, annotation, and external databases

The model/build metaphor extends throughout the system, such that the human reference sequence is represented as a model in the system, with each build from the Genome Reference Consortium being represented as a GMS build of that model. External data sets from sources such as dbSNP, UCSC, and Ensembl are also tracked and versioned with the model/build process. When reference genomes or annotation data sets are updated, models of individual genomes know that their inputs are no longer current, and can be rebuilt to reflect the latest data from the community. Annotation of genome models for individual subjects is typically a product of crossing the annotation of the reference genome with the variants found in the individual. Tools such as VEP [54] (the Ensembl Variant Effect Predictor) are integrated into the GMS, as well as a custom annotator that has been tuned to perform a similar task on somatic variants from cancer tumors. For pipelines processing RNA-seq data, the annotation build supplied is also used during alignment to assist in aligning across introns.

Cohorts

For analysis of a cohort, there is often one model for each member, producing conclusions about each genome in isolation, with an additional model for the entire cohort. The processing profile for the cohort describes how to analyze the initial models and draw further conclusions about the cohort as a unit (**Box 1**). In many cases, the cohort-level analysis draws directly from primary data behind the original models, or their intermediate results. For instance, a pedigree-aware variant detector may go back to the original alignments from members of a family rather than merely converge variant calling results from the individuals (See **S12 Fig**).

Subsystem - sequence alignment

One primary GMS subsystem is the alignment API which supports a variety of aligners, including DNA aligners such as BWA [15], Bowtie [55], and Maq [56]. RNA aligners currently include TopHat [16], and STAR [57]. New aligners may be added using the Tools Tree described above. The GMS alignment API leverages a sequence transformation (SX) API, allowing the user to write components that trim, filter, or subsample incoming instrument data in any order or combination. Filters may operate on individual reads or on read pairs. Each alignment module produces an "alignment result" data product with a standard API, and converts alignments into BAM format. The resulting BAMs are position-sorted, and made complete with indexes, flagstats, and the results of ‘Samtools fixmate’ to ensure that they meet common expectations across the system.

Subsystem - variant detection

Another subsystem exists in the GMS for handling variant detectors, where ‘variant’ refers to single nucleotide as well as larger structural and copy-number differences from the reference genome. The production of high-quality variant detection results often involves multiple tools and a series of in-silico validation steps to confirm findings when the tools are tuned for high sensitivity.

The GMS variant detection API includes a simple domain-specific language for specifying how variant detection should occur. Each type of variant detection has a formula specifying tool versions and parameters, and boolean logic to indicate the pattern of union, intersection, and filtering to employ for final results. The system has a single operation type of "variant detection" which takes these descriptions, composes a workflow, produces intermediate results, and ensures that the final product is a single VCF file per variant type (**Fig. 3**). Each step in a variant detection workflow will attempt to re-use previously generated results where all up-stream processing is identical, and then only executes the tools unique to that workflow.

Adding a new variant detector involves adding a module into the tool tree, described above under ‘tool tree and API’, and installing the detector in the standard way, as described under ‘Packaging’. The module in the tool tree will prepare data from the GMS to launch the detector, and will convert detector native output into the VCF form expected by the system.

Modules used to filter variants can vary greatly in complexity. The most complex tools, such as the TIGRA-SV [58] filter for structural variants, can go so far as to extract reads from the alignment BAM, generate miniature assemblies, align with alternate aligners to validate the assemblies, and then use conclusions from those alignments to pass or fail a variant. The GMS supports creation and maintenance of arbitrarily complex filters.

Experimental pipelines under development

Additional pipelines, also developed at The Genome Institute but not yet fully supported in this release, can be used at the analyst’s discretion. These include *de novo* assembly, gene prediction, somatic validation, and small RNA detection. Pipelines are also under development for cross-cohort analyses of cancer and inherited diseases.

Software testing

Each day, pipelines released with the GMS are systematically tested to verify that the output is consistent and that pipeline conclusions are insulated from stochastic effects. If, in a given pipeline step, results for the process are found to vary when using the same inputs, the responsible pipeline development team at The Genome Institute will be alerted that the pipeline in question is not deterministic.

Hardware, software and personnel requirements

To install the GMS a minimum of a single dedicated Ubuntu 12.04 workstation is required. All software used by the GMS built-in pipelines can be downloaded during installation as follows:

```
git clone https://github.com/genome/gms.git
```

The default installation configures the host as a single-node OpenLava cluster, that can be expanded arbitrarily. Future versions of the GMS will be compatible with SGE, a primary target for the next release. It is recommended to have at least 64 GB of RAM on some hosts in the cluster. Some steps request 4 CPUs per step, and will queue if the necessary number/specification of hosts are not available. The GMS as used at TGI takes advantage of a cluster of approximately 4,000 cores. While the GMS makes a major portion of analysis as simple as running a few commands, interpretation of the data typically requires bioinformatics expertise. Further, extending the system to use a cluster is best done in the hands of a researcher with Linux administration capabilities.

Development Environment

Because GMS software targets Ubuntu Linux 12.04, development in that environment, or on a VM in that environment, is recommended.

The GMS has been developed using the agile methodology of software development. This approach involves close interactions with the users of the software, short development cycles, pair programming, frequent communication (both between developers and with the users), and test-driven development. There is a concerted effort to develop tools and pipelines as a collaboration between analysts, software engineers, and research computing specialists. This enables tools to progress rapidly to usability in our analysis pipelines and affords the ability for significant code and pipeline optimizations.

Continuous integration is a cornerstone of the GMS development process, and is facilitated by the structure of the software and the database. Each build tracks the exact "snapshot" of software used by the system, allowing for the system code to be updated while long-running builds are still in-progress on older versions of the code. The GMS can be readily used with the Jenkins Continuous Integration server (http://jenkins-ci.org/) and should readily adapt to other similar products.

Packaging

The packaging strategy behind the GMS (described below) ensures that different versions of a tool can exist at the same time on the same machine. Often, the exact tool to use at a given point in the workflow is itself a parameter in the processing profile (**Box 1**). A researcher can attach to the GMS APT server (http://apt.genome.wustl.edu) and receive updates to packaged versions of the software used by GMS pipelines through standard methods on Ubuntu/Debian Linux distributions.

Extendibility

While pre-configured and optimized pipelines provide ease of use, their presence in no way limits the ability of the GMS to allow experimentation and extension. The GMS provides an application programming interface (API) to plug in new tools or implement them from scratch, to design and implement entire new pipelines/processing profiles, and to perform in silico experiments. For example, one could explore the effect of varying alignment parameters, base calibration, variant filters, localized assembly, etc. on the false positive and false negative rate of an entire analysis pipeline. These capabilities of the GMS help to solve the long-standing problem of supporting multiple computational environments, not just for a single tool or pipeline, but for nearly all tools and pipelines.

Indirect subjects (Transcriptome, epigenome and microbiome models)

The conclusions about the subject present in the data set produced by a given build typically relate directly to the genome sequence. Some models take inputs that are *indirectly* related to the genome. For example, cDNA reads from RNA, model the transcriptome as it relates to the genome. Similarly, bisulfite-treated reads might model the epigenome associated with the genome, and data collected from a microbiome sample might be considered the genome of the subject in only the broadest sense of the term. These all fall into the "genome" modeling system, as they still center around a genome as the underlying subject.

Workflow management

The steps in a build process are managed by a workflow system that sits at the heart of the GMS. A graph of steps is generated, with edges representing dependencies between steps. As soon as all dependencies are available for a step, the workflow server submits a single job to the associated compute cluster, with resource requirements computed according to the inputs. As jobs start, run, and complete or fail, the GMS database is updated, allowing the analyst to monitor progress, examine logs, and inspect intermediate results. A web interface (**S14 Fig**) and command-line interface (**S1 Fig-S2 Fig**) are available for build tracking. The workflow system in the GMS has several useful characteristics including shortcutting to avoid reproducing data when it has been created previously, transparent nesting, and auto-parallelization when the number of outputs of a step varies. The workflow system distributes jobs to a compute cluster. For a standalone installation, a one-node cluster is installed that can be expanded later. The default installation uses OpenLava (http://www.openlava.org), an open-source fork of Platform LSF (http://www.platform.org).

Database and disk management

Data about models, processing profiles, builds and subjects are stored in a scalable relational database management system (RDBMS), built on PostgreSQL 9.2 (http://www.postgresql.org). This data is accessible from an API with an implementation in both Perl (UR) (http://search.cpan.org/~sakoht/UR-0.30/lib/UR.pm) and Ruby (ActiveRecord) (http://rubygems.org/gems/activerecord). Detailed analysis results are represented in files as defined by the bioinformatics community and are stored in a fashion that is built to scale with computational tools. For example, Illumina DNA sequence reads are stored in BAM format, as are alignments. Variant detection results are stored in VCF files with block-gzip compression to allow querying without full decompression via Tabix [14]. The GMS includes a Disk Allocation System that stores data about each slice of disk used in the RDBMS, along with information about the owner of the data. The latter is typically a given build, or a "software result" that can be shared by builds (**Box 1**). Processes that require disk request an appropriate amount beforehand, and then re-size their allocation after processing completes to handle differences between expected and actual disk usage. The disk allocation system allows administrators to re-locate data as required without interrupting processing.

Federation

The GMS is “federation ready”, such that a researcher working on one machine could attach the GMS of another researcher, and perform analysis on these data, if permissions were granted on either side. The GMS uses globally unique identifiers for

all data, whether in a database table or on a file system. The data shared can be actively copied or virtually "mounted", allowing for the transfer of data to occur in a fashion that does not consume resources prematurely. This is particularly useful for cases in which the final compute will occur later, at a more remote host.

Workflow feature details

Shortcutting
Most significant data sets are generated only once in a given GMS model for a given set of inputs and parameters. Steps that repeat a deterministic process that have already been accomplished by another pipeline, or earlier in the same pipeline run, or during a previous build of the current model, will attach the previous results instead of reproducing them. Locking around this process prevents several pipelines from attempting to create the same set of data. Only one will actually perform the work, and all of the builds in question will share the result when it is ready. Each of these intermediate "software result" entities help the system operate like a large "make" (http://www.gnu.org/software/make/) system (**Box 1**). This system uses pessimistic concurrency (ISBN: 0470128720) intentionally (using locking instead of transaction repetition) to optimize use of CPU and disk.

Auto-parallelization
Some steps in a pipeline produce a varied number of outputs. A subsequent step can be configured to auto-parallelize on the basis of one of its inputs. This allows a workflow to be uncertain up to a given point about the exact graph that will be used, and to allow that graph to dynamically expand. The most basic example of this is at the beginning of pipelines that perform alignment. Though it is declared as one step in the pipeline, it automatically expands the graph based on the number of units of instrument data supplied. The subsequent step, performing alignment merging, and marking of duplicates, is configured to wait for all of its predecessors, and to handle a dynamic number of inputs.

Nesting
When a given step in a workflow creates its own workflow and initiates execution of it, that workflow recognizes itself as subordinate to the step that initiated it, and visualization of the parent workflow will include child steps. This allows components to compose in a scalable way. For example, basic pipelines that perform alignment and variant detection have a single step for variant detection. The variant detection component, however, creates a subordinate workflow for each tool that must be run. Further, the Pindel indel detection component internally is built to perform per-chromosome parallelization. The Breakdancer structural variant detector benefits from

per-chromosome parallelization, plus an additional step to detect cross-chromosome translocations. **Fig. 3** shows this in detail.

An upcoming release will replace the existing Workflow system with a new, Petri-net based system. The current system scales well for analyses shown here, but fails at workflows with tens of thousands of tasks. The new system should also relieve the GMS of dependency on OpenLava, the open-source fork of Platform LSF, and allow for use on SGE, PBS, or other job scheduling systems commonly used on compute clusters. The existing GMS leverages the ability to “mount” data (virtually attach remote data as though it were local disk), but expects any given GMS installation to mount or “auto-mount” all disk used within the particular instance. While this has scaled to 15 PB of data for TGI, it is suboptimal in many regards, and would hopefully be replaced with a system to do just-in-time data attachment.

Stability and security of analysis results

The GMS has many features that attempt to ensure the provenance of results as well as their stability over time. Prominent among these is the GMS’s use of immutability. Data that are necessary as input to other analysis processes are never deleted under normal operation of the GMS. They might be superseded by an updated version, but the old version will remain in case it is already being used by other downstream analyses. For example, GMS ‘builds’ are never deleted, even if they are ‘abandoned’, this is noted in the database, but the actual underlying data remains in place. You can delete a GMS model, but only if no builds exist for that model. Furthermore, files are compartmentalized as 'software results', even if an analysis is updated, the old results are not deleted or over-written. The GMS enables a reversible archiving process to manage disk usage but during normal functioning of the GMS nothing is ever deleted or destroyed other than temporary files during analysis runs that are not meant to be stored.

As for user security, within a trusted group you can use the same GMS. Between groups where trust is less complete, you would use different GMSs, and allow them to interact. Because the GMS leans on standard Unix protocols and security, it is as secure as those systems, and can be secured by non-bioinformatics technical staff.

GMS principles and their relation to internationals standards of software design

The ISO quality standards (ISO/IEC 9126 and subsequent ISO/IEC 25010:2011) attempt to define software quality across six dimensions: functionality, reliability, usability, efficiency, maintainability, and portability. This system, like most production systems, has room for improvement in each of these areas, but we believe it makes a

substantial showing in each as well. Following are details on the system's status in each respect.

Functionality

The GMS has a fairly broad mandate in the genomics space: to handle all large-scale data processing for the variety of tasks involved in large-scale genome analysis. Because this involves processing large volumes of data consistently for large projects, but must also support methods development, it aims to be both a production and a research tool. It does not, however, step into the space of automated exploration, genetic algorithms, or self-tuning, though its structure is not opposed to it. It is also not built to provide data to naive users, or to provide an interface for public/untrusted users.

Reliability

Data flagged as successful must be trusted if it is to be built upon. In the context of the GMS "reliability" means being certain that things will fail cleanly when they cannot be trusted with high certainty. Most software results are created in a staging area to ensure that a failure even at the very end, causes all incomplete work to be discarded. Granularization of the tasks mitigates the loss of compute effort. Defensively written generic code around the processes allows the system to rely slightly less on the careful diligence of the programmer, though as always, this is still primary in good software. Many take a snapshot of new results and compare to previous results in a very broad way, allowing for a test to catch problems the author did not foresee. This is a maintenance burden as the test result must be re-verified for some incidental changes, but it provides significant confidence to the researchers.

Usability

The GMS is aimed at the genomics power-user. As such, it allows an organization with genomics data experts to operate at scale. It would not be considered usable by web-only users, or researchers uncomfortable with the underlying tools it wraps. It does, however, encapsulate some of the logistical complexity of processing genomes. Features such as dynamic tab-completion for thousands of commands ensure that the target user, a bioinformatics specialist, is able to operate in agile fashion.

Efficiency

The core of the GMS result generation layer aims to follow the "DRY" (Don't Repeat Yourself) principle systematically. Any code run in the exact same way on the same data need run only once, and will be found by any other pipeline with a processing path that overlaps it. There are other approaches which create a different kind of efficiency, such as streaming, wherein data is transferred between steps without saving it. The GMS makes a trade-off to have reusability of intermediate units, and to allow the

endpoints to be asynchronously available. A streaming approach would avoid saving those units entirely, but would require the endpoints to be active simultaneously, and would contend with the associated fragility increase. In addition to avoiding repeating work, each process can identify the resources it will consume dynamically, allowing the compute cluster to optimally fit jobs on worker nodes. Many steps that use multi-threaded underlying components are aware of this, and will consume a sufficient portion of a host node to use that feature as the component author specifies.

Maintainability

The GMS is agreeable to parallel development, and the ability to do this is the foundation of maintainability. One engineer can improve the way all builds process, while another adds features to a specific pipeline, and their results can operate in concert. A thorough testing framework, along with a clean separation of concerns, is foundational to this aspect of the system. Versioning of data sets is also a key part of maintainability. Without this, a software system calcifies around its first implementation. Parallel development is fostered by an organized software tree built on the "principle of least surprise". With a standard place to put things, one knows where to look to find prior work, and knows where to put the next contribution.

Portability

The GMS is a full system image, it is not meant to operate as an application installed in an unknown environment. It is, however, built to interact with other systems as an appliance, and integrate as seamlessly as possible through standard internet protocols.

Tissue culture and nucleic acid isolation of HCC1395/HCC1395BL

HCC1395 cells were purchased from the American Type Culture Collection (ATCC, Manassas, VA). The only data released with this manuscript are derived from a cell line. This material is considered by our institute to be 'non-human' research materials. The cells were grown at 37°C in 95% O2–5% CO2. HCC1395BL cells were cultured in Iscove's Modified Dulbecco's Medium (IMDM) with 20% fetal bovine serum (FBS), and 1% penicillin/streptomycin (P/S). HCC1395 cells were cultured in RPMI with 10% FBS, and 1% P/S. Cells were minimally passaged from time of purchase to reach desired cell numbers. Genomic DNA and RNA were isolated from cells of the same passage. RNA was isolated using RNeasy Mini Kit (Qiagen, Valencia, CA) following the manufacturer's instructions with the recommended on column DNase I (Qiagen) digestion. Genomic DNA was isolated with the DNA Blood and Tissue Kit (Qiagen) with a RNase A digestion (40ug/uL). All RNA and DNA were eluted in water.

Whole genome sequencing of HCC1395/HCC1395BL

For each whole genome shotgun library, 350 ng DNA was fragmented in 5x DNA Terminator End Repair Buffer (Lucigen, Middleton, WI) using the Covaris S2 and micro-TUBEs (Covaris, Woburn, MA) using the following settings: volume = 50 µL, temperature = 4 °C, duty cycle = 5, intensity = 4, cycle burst = 200, time = 90 seconds. The fragmented ends were converted to blunt ends by adding DNA Terminator End Repair Enzyme and following the manufacturer's protocol. The blunt ended DNA was then purified using using MinElute columns per manufacturers protocol (Qiagen). DNA was eluted with 32 µL 10 mM Tris-HCl (pH 8.0). A 3' A overhang was added to the blunt ended fragments by treating with 15 units of Klenow Fragment 3'->5' exo- and 200 nM dNTP mix (New England BioLabs, Ipswich, MA) for 30 minutes at 37 °C.

Each sample was then ligated with 2.5 µL of a 9 µM stock of Illumina TruSeq LT adapters. The ligation reactions were accomplished using 5,000 units of T4 DNA ligase (New England BioLabs, Ipswich, MA). We purified each adenylation reaction using MinElute columns and DNA was eluted with 20 µL 10 mM Tris-HCl (pH 8.0). Next, we ligated the Illumina TruSeq LT adapters, A03 (5' TTAGGC) and A04 (5' TGACCA), to DNA samples H_NJ-HCC1395_T (tumor) and H_NJ-HCC1395_BL (blood normal), respectively. The reaction set-up was as follows: DNA was suspended in 1x Quick Ligation Buffer (NEB), 450 nM Illumina TruSeq adapter, and to minimize adapter-dimer formations, we added 5,000 units of Quick Ligase (NEB) and incubated the reaction for 15 minutes at 25 °C.

Each ligated DNA sample was purified using MinElute columns and DNA was eluted in 10 µL 10 mM Tris-HCl (pH 8.0). Following the Illumina standard protocol, we PCR-amplified our ligated libraries with minor modifications. To prevent excessive over-amplification during the PCR, we cycle optimized our libraries. Each 50 µL reaction included 1 µL of eluted ligated DNA, 1x Phusion PCR Master Mix, and 200 nM each forward primer and reverse primer pair: Forward 5' – AATGATACGGCGACCACCGAGATCTACACTCT and Reverse 5' – CAAGCAGAAGACGGCATACGAGAT. The reactions were cycled as follows: 9830sec, Cycle – 9,810 seconds, 6,530 seconds, 7,230 seconds, and after cycles 4, 6, and 8, the program was halted and a 5 µL aliquot was collected. After the final collections at 8 cycles, each cycle amplification product was evaluated on a 1.1% agarose Flash Gel (Lonza). This qualitative assessment illustrated the cycle number where over-amplification occurred as observed by shifts in DNA mobility on the gel. Based on the qualitative assessments, we determined our optimal cycle number for both H_NJ-HCC1395 and HCC1395_BL WGS libraries to be 6 cycles.

We performed eight amplification reactions as described above using 1 µL template per reaction for 6 cycles in the PCR. Post PCR, we purified each library by (1) pooling the 8 H_NJ-HCC1395_T reactions, and (2) pooling the 8 HCC1395_BL reactions. Each library pool was purified with MinElute columns and fractionated on the Caliper LabChip XT using the DNA 750 chip (Perkin Elmer, Hopkinton, MA). For both libraries, H_NJ-HCC1395_T and H_NJ-HCC1395_BL, we collected three unique fractions: 375 bp, 475 bp, and 650 bp. Each fraction was assessed for concentration and size to determine library molarity using the Qubit Fluorometer Quant-iT dsDNA HS assay (Life Technologies, Grand Island NY) and the Agilent BioAnalyzer DNA 1000 Assay (Agilent Technologies, Santa Clara, CA), respectively.

To accurately quantify each library, we diluted each fraction to 5 nM and determined molarity using 425 bp qPCR standards serially diluted 10-fold from 20pM to 0.0002 pM supplied in the KAPA SYBR FAST qPCR Kit (KAPA Biosystems, Woburn, MA). Based on the qPCR results following the manufacturer's protocol, each library fraction was diluted to 2 nM and processed for Illumina sequencing.

Exome capture and sequencing of HCC1395/HCC1395BL

To prepare libraries for exome capture, we fragmented 500 ng H_NJ-HCC1395 _T and H_NJ-HCC1395_BL DNA as described above for the WGS libraries with a minor modification to the shearing parameters and ligated Illumina TruSeq LT adapters to each sample with index sequences, ATCACG and CGATGT, respectively (Shearing Parameters: volume = 50 µL, temperature = 4 °C, duty cycle = 20, intensity = 5, cycle burst = 500, time = 120 seconds). Each library was PCR-optimized as described above requiring six cycles. Again, we performed eight amplification reactions for each library, pooled each reaction, and purified the amplified library with MinElute columns. Library yields were 1,554 ng and 1,090 ng for H_NJ-HCC1395_T and H_NJ-HCC1395_BL, respectively. Each library was size-fractioned using solid-phase reversible immobilization using Ampure paramagnetic particles activated with carboxyl functional groups (Beckman Coulter, Inc.). To obtain a ~300-500 bp library fraction prior to going into targeted-enrichment, we used a 1:0.6 sample to AMpure bead ratio to which fragment sizes ~500 bp and below are retained within the supernatant. We then combined the supernatant with 0.9 X volumes of beads to which fragment sizes >300 bp are bound to the paramagnetic particles. The resulting supernatant was discarded, beads washed, and the size-fractioned capture libraries were eluted and quantified.

For target-enrichment, we pooled 500 ng of each PCR-amplified, SPRI-fractioned library and added 5 µg Human Cot-1 DNA, 1 mM adapter blockers, and SeqCap EZ Human Exome Library v3.0 capture probes (Roche NimbleGen, Madison, WI.). Each

hybridization reaction was denatured with heat and allowed to reassociate at 47 °C for 72 hours. Hybridized library fragments and capture probes were bound to Dynabeads M-270 Steptavidin-coated paramagnetic particles (Invitrogen) and stringently washed with provided buffers. Enriched single-stranded (ss) DNA library fragments were denatured from the capture probes with 0.125 N NaOH, neutralized with 1 M Tris-HCl (pH 8.8), and recovered from solution using a 1.5:1.0 Ampure bead-to-sample ratio. The enriched ssDNA library fragments were eluted and recovered in 20 µl 10 mM Tris-HCl (pH 8.0).

Each sample was amplified in the PCR using 20 µl of enriched ssDNA library fragments, 1X Phusion PCR Master Mix, and 200 nM each forward primer and reverse primer pair: Forward 5' – AATGATACGGCGACCACCGAGATCTACACTCT and Reverse 5' – CAAGCAGAAGACGGCATACGAGAT. Each enriched library was assessed for concentration and size to determine library molarity using the Qubit Fluorometer Quant-iT dsDNA HS assay (Life Technologies, Grand Island NY) and the Agilent BioAnalyzer DNA 1000 Assay (Agilent Technologies, Santa Clara, CA), respectively. Samples were diluted to 2 nM prior to Illumina sequencing.

RNA sequencing of HCC1395/HCC1395BL

Isolated total RNA from sample(s) was DNase-treated using 2 units of TURBO DNase (TURBO DNA-free Kit, Life Technologies, Grand Island, NY). The DNase-treated RNA samples were concentrated using a ratio of 1:1.8 sample to RNAClean XP beads (Beckman Coulter, Indianapolis IN), and RNA mass was determined using the Qubit Fluorometer and the Quant-iT RNA Assay (Life Technologies, Grand Island, NY). We used 1 µg of DNase-treated total RNA and selected poly(A) RNA using the Dynabeads® mRNA DIRECT Micro Kit (Life Technologies, Carlsbad CA). Isolated poly(A) RNA was suspended in 10 µl nuclease free water at a final concentration of 2.3 ng/µl and diluted to 1 ng/µl. Generation of cDNA used 1 ng poly(A)-selected RNA as input into NuGEN's Ovation RNA-Seq System V2 (NuGEN, San Carlos, CA). The generated cDNA was assessed for concentration (93.5 ng/µl; total yield: 2,711 ng). 1000 ng of the NuGEN generated cDNA was fragmented in 5X DNATerminator End Repair Buffer (Lucigen, Middleton, WI) using the Covaris S2 and micro-TUBEs (Covaris, Woburn, MA) using the following settings: volume = 50 µL, temperature = 4 °C, duty cycle = 5, intensity = 4, cycle burst = 200, time = 90 seconds. Illumina TruSeq LT adapter AD05 was used in library construction and amplified as outlined for WGS requiring seven PCR cycles (8 reactions). We used Ampure paramagnetic particles to size select ~300-500 bp cDNA library fragments as described for exome capture. The size-selected cDNA library was diluted to 2 nM based on KAPA qPCR results and processed for subsequent Illumina sequencing.

Analysis of HCC1395/HCC1395BL data

Five lanes of Illumina paired 2x100 bp data were produced for whole genome sequencing of HCC1395, three lanes for whole genome sequencing of HCC1395BL, one lane for the exome data for both HCC1395 and HC1395BL, and one lane for HCC1395 and HCC1395BL transcriptomes.

Genotype calls were calculated from the Illumina Infinium microarray data (see above) using the GMS genotype microarray processing profile: 'infinium wugs' (2575175). Alignment of WGS and Exome reads was performed using the GMS processing-profile: 'Nov 2011 Default Reference Alignment' (2635769). This version of the pipeline uses BWA [15] version 0.5.9 for alignment with default parameters except for the following: '-t 4 -q 5'. Alignments were performed on a lane-by-lane basis and then merged with Picard. All alignments were against build37 (hg19) of the human reference genome. After reference alignment, germline variant calling was performed on each sample independently using samtools and filtered using custom filters internal to the GMS. Genotypes from the sequence data were compared back to genotypes from the corresponding genotype microarray results for each sample. A combination of SNVs, small insertions and deletions (indels), CNVs, and SVs were detected by analyzing the WGS tumor and normal, and again independently analyzing the exome tumor and normal. Somatic variant detection from WGS data occurred as shown in **Fig. 3**, using the GMS processing profile: 'Oct 2012 Default Somatic Variation WGS' (2762562). Somatic variant detection from Exome data was performed using the GMS processing profile: 'Oct 2012 Default Somatic Variation Exome' (2762563). Somatic SNV detection was performed in this pipeline using a combination of Strelka [46], VarScan [47] and SomaticSniper [48]. Somatic small indels were detected by a combination of Pindel [49], GATK [59], Varscan, and Strelka. Annotation of variants was performed using the GMS transcript variant annotator against a GMS 'annotation' build based on Ensembl v67 transcripts [60]. SNVs and indels were compared against a GMS 'previously discovered variations' build based on dbSNP version 137. SNVs and Indels were also compared against Cosmic v65.1 variants mutation data [39]. Copy number variants were detected by 'cnv-hmm' and structural variants were detected by a combination of BreakDancer [43] and SquareDancer (http://tvap.genome.wustl.edu/tools/squaredancer/). RNA-seq data analysis was performed using the GMS processing profile 'October 2012 Default Ovation V2 RNA-seq' (2762841). RNA-seq data for both the tumor and the normal was aligned with TopHat [16]. Initial expression data was generated using Cufflinks on a per-sample basis, and also with HTSeq [18]. Fusion detection results were produced with ChimeraScan [45] for later comparison with SV detection results from BreakDancer [43].

The WGS somatic variation genome model, along with the exome somatic variation model, and the independent tumor and normal RNA-seq models were used as inputs to the integrative MedSeq model, as shown in **Fig. 4**. The MedSeq (aka ClinSeq) pipeline produces 10 directories of data, with a total of approximately 2,000 files and images (a sampling of which are provided in the results). Results include convergence between WGS, exome and RNA-seq data, differential expression measurement, extensive annotation with public databases, and also annotation with DGIdb [11], suggesting hypotheses for drug target investigation. The output of the MedSeq pipeline is used to prepare an overview for clinicians on high-priority research cases but is also a convenient starting point for cancer biomarker survey studies.

Supplementary References

54. McLaren W, Pritchard B, Rios D, Chen Y, Flicek P, Cunningham F. Deriving the consequences of genomic variants with the Ensembl API and SNP Effect Predictor. Bioinformatics. 2010;26(16):2069-70. Epub 2010/06/22. doi: 10.1093/bioinformatics/btq330. PubMed PMID: 20562413; PubMed Central PMCID: PMC2916720.
55. Langmead B, Trapnell C, Pop M, Salzberg SL. Ultrafast and memory-efficient alignment of short DNA sequences to the human genome. Genome biology. 2009;10(3):R25. Epub 2009/03/06. doi: 10.1186/gb-2009-10-3-r25. PubMed PMID: 19261174; PubMed Central PMCID: PMC2690996.
56. Li H, Ruan J, Durbin R. Mapping short DNA sequencing reads and calling variants using mapping quality scores. Genome research. 2008;18(11):1851-8. Epub 2008/08/21. doi: 10.1101/gr.078212.108. PubMed PMID: 18714091; PubMed Central PMCID: PMC2577856.
57. Dobin A, Davis CA, Schlesinger F, Drenkow J, Zaleski C, Jha S, et al. STAR: ultrafast universal RNA-seq aligner. Bioinformatics. 2013;29(1):15-21. Epub 2012/10/30. doi: 10.1093/bioinformatics/bts635. PubMed PMID: 23104886; PubMed Central PMCID: PMC3530905.
58. Chen K, Chen L, Fan X, Wallis J, Ding L, Weinstock G. TIGRA: A targeted iterative graph routing assembler for breakpoint assembly. Genome research. 2014;24(2):310-7. Epub 2013/12/07. doi: 10.1101/gr.162883.113. PubMed PMID: 24307552; PubMed Central PMCID: PMC3912421.
59. DePristo MA, Banks E, Poplin R, Garimella KV, Maguire JR, Hartl C, et al. A framework for variation discovery and genotyping using next-generation DNA sequencing data. Nature genetics. 2011;43(5):491-8. Epub 2011/04/12. doi: 10.1038/ng.806. PubMed PMID: 21478889; PubMed Central PMCID: PMC3083463.
60. Flicek P, Ahmed I, Amode MR, Barrell D, Beal K, Brent S, et al. Ensembl 2013. Nucleic acids research. 2013;41(Database issue):D48-55. Epub 2012/12/04. doi: 10.1093/nar/gks1236. PubMed PMID: 23203987; PubMed Central PMCID: PMC3531136.
